# Phytochemistry, Pharmacology and Quality Control of Xiasangju: A Traditional Chinese Medicine Formula

**DOI:** 10.3389/fphar.2022.930813

**Published:** 2022-06-23

**Authors:** Siyuan Wu, Hua Luo, Zhangfeng Zhong, Yongjian Ai, Yonghua Zhao, Qionglin Liang, Yitao Wang

**Affiliations:** ^1^ Macau Centre for Research and Development in Chinese Medicine, Institute of Chinese Medical Sciences, University of Macau, Taipa, Macau SAR, China; ^2^ State Key Laboratory of Quality Research in Chinese Medicine, Institute of Chinese Medical Sciences, University of Macau, Taipa, Macau SAR, China; ^3^ Department of Chemistry, Center for Synthetic and Systems Biology, MOE Key Laboratory of Bioorganic Phosphorus Chemistry and Chemical Biology, Beijing Key Lab of Microanalytical Methods and Instrumentation, Tsinghua University, Beijing, China

**Keywords:** Xiasangju, *Prunella vulgaris* L., *Morus alba* L., *Chrysanthemum indicum* L., phytochemistry, pharmacology, quality control

## Abstract

As a traditional Chinese herbal formula, Xiasangju (XSJ) is widely used in China for antipyresis and influenza treatment. However, XSJ still fails to have a comprehensive summary of the research progress in the last decade. This review summarizes the advanced research on the extraction process, phytochemistry, pharmacological activity, and quality control of XSJ. Current research mainly focuses on quality control and the pharmacological effects of single herbs and active ingredients, but many pharmacological mechanisms of the formula are unclear. The development of active ingredients reflects the active characteristics of triterpenes, phenolic acids and flavonoids, but the hepatotoxicity of *Prunella vulgaris* L. has not been taken into account. XSJ has extensive historical practical experiences, while systematic clinical trials remain lacking. Therefore, it is necessary to study the active ingredients and define the mechanisms of XSJ to develop multiple applications, and further studies on the dose range between its hepatoprotective activity and hepatotoxicity are necessary to improve the safety of the clinical application. In this review, the current problems are discussed to facilitate the reference basis for the subsequent research on the development of XSJ and future application directions.

## 1 Introduction

Xiasangju (夏桑菊, XSJ), a traditional Chinese herbal formula, consists of Prunellae spica (*Prunella vulgaris* L., PV), Folium mori/Mulberry Leaf (*Morus alba* L., MA), and Chrysanthemi indici flos (*Chrysanthemum indicum* L., CI), derived from a classic formula “Sangju Yin (桑菊饮)” in the monograph entitled “Analysis of Warm Diseases (Wen Bing Tiao Bian)” by Wu Jutong, a famous febrile disease scientist in the Qing Dynasty (1798 AD) (Xu and Wei, 2013). It has been a common herbal tea formula for a long time in Guangdong, China. Based on more than 200 years of clinical practice and transmission, the modern pharmaceutical process has refined it into XSJ formulations, including XSJ granules, XSJ capsules, XSJ oral liquid, XSJ effervescent tablets, etc. They mainly play the roles of clearing liver fire to improve eyesight, resolving exterior, clearing lung-heat for arresting cough, removing dampness, and diffusing impediment, and relieving sores and toxins, and are usually used for treating the wind-heat type of cough, whose symptoms include slight fever, cough, dry lips, failing to expel phlegm smoothly, sore throat (Yao et al., 2017a; Chinese Pharmacopoeia Commission, 2020). Recent studies have shown that it also has pharmacological activities such as antioxidant, anti-tumor, antidiabetic activity, hepatoprotective, and renoprotective effects (Ma et al., 2011; Yu et al., 2011). Some health products, such as XSJ health tea is regarded as a refreshing drink and XSJ lozenges contribute to throat moistening.

The prescription of XSJ granules recorded in Pharmacopoeia of the People’s Republic of China (Ch.P) 2020 is composed of 500 g of PV, 80 g of CI, and 175 g of MA (Chinese Pharmacopoeia Commission, 2020). The formula is based on “one Sovereign (Jun) two minister (Chen)” of the principle of traditional Chinese medicine (TCM) compatibility. PV is regarded as “Sovereign (Jun) medicinal,” which has the functions of clearing fire, brightening eyes, reducing swelling and dispersing nodules, and is suitable for treating redness and swelling of eyes, headache, and vertigo, scrofula, canker sores, enlarged thyroid gland, lymph nodes, breast hyperplasia, and hypertension (Chinese Pharmacopoeia Commission, 2020). CI and MA are the “minister (Chen) medicinals.” CI clears heat-toxin, while MA resolves exterior, clears lung-heat, and moistens dryness, and removes liver fire for improving eyesight. The combination of CI and MA has the effects of liver cleansing and detoxification, anti-inflammation and antibacterial, which complements each other and augments the major action of PV. XSJ granule is the 11th category of TCM formula included in the “Drug Standard of the Ministry of Health of the People’s Republic of China” (Book XV) (National Health Commission of the People’s Republic of China, 2000). The quality standard of XSJ granule has been established by high performance liquid chromatography (HPLC), and the content of rosmarinic acid in PV is determined to be not less than 2.5 mg/bag. However, quality control of the XSJ granule needs to be continuously improved.

The application of XSJ is very popular in China, as an herbal drink in summer, because of its mild, and long-lasting effects. There are no significant side effects like drowsiness and weakness of western medicines for cold and flu. In recent years, with the explosion of the herbal tea beverage market, XSJ granule has been increasingly known by more people relying on their exact efficacy on wind-heat and its cooling taste, gradually exporting to countries outside of China. XSJ granule market has covered throughout Guangdong and South China, and the products are sold to Hong Kong, Macao, the United States, Canada and other countries or regions. In addition, 107 improved formulations of XSJ in the form of granules, tablets, and capsules have received marketing approval from the State Food and Drug Administration of China, and four products exported abroad belong to granule (National Medical Products Administration, 2022).

According to the Global Biodiversity Information Facility database (https://www.gbif.org/) **(** Supplementary Figure S1), georeferenced records of the three herbs up to 2022 indicate a remarkable regional distribution and abundant resources (Gbif | Global Biodiversity Information Facility, 2022). PV, the common self-heal, selfheal, or heal-all, is an herbaceous plant in the mint family Lamiaceae. There are 10 primary species of PV worldwide, with a total of 7 subspecies, 1 variety, and 2 forms recorded. It was widely distributed in the temperate regions and tropical mountains of Europe and Asia, south-eastern Australia, and North America (Gbif.Org., 2022c). In addition, the primary species of MA recorded 3 varieties and 1 form. MA mainly distributes in the temperate and tropical regions, including South Europe, North America, East and Southeast Asia, south-eastern Australia, and some parts of Africa (Gbif.Org., 2022b). CI was distributed in Eurasia, North America, and other regions, and widely distributed in China, Japan, Korea, and India, with a total of 12 recorded varieties (Gbif.Org., 2022a; Encyclopedia of Life, 2022). The distribution of the three herbs in East Asia relates to their Chinese application patterns. PV has long been used as a self-healing medicine in Europe and the United States (Wagner et al., 2020), mainly used for antifebrile, immunoregulation, anti-inflammation, treating breast disorders, especially for relieving sore throats and fevers, and facilitating wound healing (Bekut et al., 2018). The differences in the folk application habits of XSJ may mainly depend on the geographical distribution of the herbs.

In recent years, the study of each herb of XSJ has been reviewed, but as a formula, the research progress of XSJ over the past decade has yet to be summarized. This review summarizes the advanced research on the extraction process, phytochemistry, quality control, and pharmacological activity of XSJ. Current research mainly focuses on the quality control and pharmacological effects of single herb and its active ingredients, but the pharmacological mechanisms of the formula are unclear. The development of active ingredients reflects the characteristics of triterpenes, phenolic acids, and flavonoids, but the hepatotoxicity of PV has not been taken into account. XSJ has extensive practical experience in TCM, but systematic clinical trials remain lacking. Therefore, it is necessary to study the active ingredients and mechanisms of XSJ to develop multiple applications. Further studies on the dose range between its hepatoprotective activity and hepatotoxicity are necessary to improve the safety of clinical use. In this review, the current problems are discussed to consolidate the reference basis for the subsequent research on the development of XSJ and future application directions.

## 2 Extraction Methods and Purification Procedures

The extraction process of XSJ is based on the aqueous extraction method, by which the three herbs are decocted in water. Then the alcohol precipitation method is used to obtain the infusion. Lin et al. (2012b) used total flavonoids, total polysaccharides, and paste yield as the index components, and used the orthogonal experimental design to select the optimal extraction process of XSJ as 12 times the amount of water decoction, and then extracted 3 times for 2 h each. At present, the extraction process of XSJ mainly focuses on increasing the content of active ingredients and retaining the aromatic and volatile active ingredients of CI. The original decoction method would lead to the destruction of the active ingredients such as chlorogenic acid and linarin by high temperatures. Therefore, Guangzhou Xingqun Pharmaceutical has developed a preparation method to extract part of CI separately by taking a small amount of CI to obtain an alcoholic extract, and then combining it with the aqueous extracts of the remaining CI, PV, and MA (Supplementary Figure S2) (Sun et al., 2008). This extraction method has been recorded in the Ch.P, and sucrose was used as an adjuvant to prepare yellow-brown colored XSJ granules. In practical production, Xiao et al. (2014) preferred the extraction process of XSJ based on the homogeneous extraction technique, and used the orthogonal test method with the relative mass per herbal extract, dry paste yield and fingerprint peak information as the comprehensive scoring indexes to guide the efficient and rapid extraction of the components in XSJ. The optimal conditions were as follows: 30% ethanol by volume, 45 times the amount, and 70°C extraction temperature.

Some studies have further applied membrane separation technology to the refinement of XSJ extracts to improve the yield of chlorogenic acid and ursolic acid. The process is carried out by inorganic ceramic membranes or organic composite membranes to refine and separate the extracts, and then the permeate is concentrated by nanofiltration or reverse osmosis to obtain the infusion. The results showed that it could effectively prevent the loss of active ingredients during the separation of impurities, and the enrichment rate was higher than that of the original alcohol precipitation process (Sun et al., 2006). Another study had also concluded that the alcohol precipitation method led to too much ethanol, which was detrimental to the safety and taste of the drug, so the extraction solution was obtained by centrifugal filtration followed by low-temperature ultrafiltration (Zhao, 2015). However, given the economic values such as the cost and efficiency of extraction, the main extraction and purification process still retain the alcohol precipitation method.

## 3 Phytochemistry

### 3.1 Physiochemical and Structural Features

The TCM formulas have various and complex components. Modern medicine methods of separation, screening and pharmacological activity research have essentially elucidated the material basis of pharmacological effects of formulas and given the scientific connotation to TCM formulas. The isolation and identification of the chemical constituents of XSJ are beneficial in advancing the scientific verification and modern interpretation of their corresponding pharmacological activities. Studies of XSJ primarily include the overall chemical composition and the change of the chemical composition before and after compatibility. Results show that rosmarinic acid from PV, chlorogenic acid from MA and CI, linarin from CI are the three main active ingredients in XSJ, which are considered as markers for quality control (Lin et al., 2013; Chinese Pharmacopoeia Commission, 2020). With the development of modern detection technology, studies have established qualitative and quantitative methods to determine the active ingredients in XSJ, focusing on quality standards and content determination. By LC-MS, NMR, and HPLC-MS, 31 compounds were identified in the ethanol-extracted XSJ spectrum. They were mainly identified as oleanolic acid 1), β-Amirin 2), ursolic acid 3), protocatechuic acid 4), caffeic acid 5), rosmarinic acid 6), salviaflaside 7), chlorogenic acid 8), caffeoylquinic acid 9), dicaffeoylquinic acid 10), kaempferol 11), quercetin 12), apigenin 13), luteolin 14), acacetin 15), diosmetin 16), astragalin 17), kaempferol 3-O-rutinoside 18), quercetin 3-glucoside 19), hyperoside 20), rutin 21), apigenin 7-O-glucoside 22), luteolin 7-O-glucoside 23), tilianin 24), linarin 25), acacetin 7-O-β-D-glucuronopyranosyl-(1→2)[α-L-rhamnopyranosyl-(1→6)]-β-D-glucopyranoside 26), diosmetin 7-O-β-D-glucoside 27), β- Sitosterol 28), β-Daucosterol 29), dotriacontanoic acid 30), n-nonadecanol 31). In the study of component analysis, there are two methods of sample preparation, one is for the ethanol extraction of the three herbs according to the prescription ratio (Liu et al., 2012; Zhou, 2012; Zhou et al., 2012; Xin and Tang, 2013), and another is for the methanol extraction of the preparation of XSJ granule (Hua et al., 2013; Lin et al., 2013; Xia et al., 2016a; Xia et al., 2016b; Wu, 2019). The extraction method combined with the different analytical methods and conditions led to differences in the results. Currently, the analysis of active ingredients in XSJ has focused on prescriptions or herbal extracts, while no quantification of active ingredients in biological samples has been reported. This review summarized the chemical components isolated and identified from the formula, as shown in (Supplementary Table S1), to provide a reference for the isolation and analysis of XSJ.

#### 3.1.1 Triterpenes

Triterpenes are a class of terpenoids whose original parent nucleus consists of six isoprene units polymerized, and exists in the plant in free form or glycosides and esters forms. PV contains various triterpenes as the major active ingredients for lowering blood pressure (Wang et al., 2019). Therefore, the study of triterpenes in XSJ on lowering blood pressure is beneficial in guiding the pharmacodynamic study. The triterpenes in PV are basically oleanolic acid 1) which is the standard control for determining saponin content (Wu, 2019). However, the isolation study of total saponins in XSJ compounds is lacking. The formula ethanol extract was extracted with petroleum ether, and then separated by silica gel column chromatography to obtain β-amirin 2), ursolic acid 3) (Zhou et al., 2012). The structures of the three isolated and identified triterpenes are shown in (Supplementary Figure S3A), where the two main components of triterpenes are oleanolic acid 1) and ursolic acid 3).

#### 3.1.2 Phenolic Acids

Phenolic acids are a large group of active ingredients in XSJ, mainly consisting of phenolic acids and other acids. Seven phenolic acids in XSJ were isolated, and most have significant biological activity (Supplementary Figure S3B). Rosmarinic acid 6) is one of the main active ingredients of PV, and salviaflaside 7) is a unique ingredient in PV corm. Rosmarinic acid 6) and salviaflaside 7) have antibacterial, antiviral, antioxidant, anti-inflammatory, immunosuppressive, and antithrombotic activities and so on (Hua et al., 2013). Phenolic acids treatment can achieve good antioxidant effects by inhibiting or scavenging free radicals. Chlorogenic acid 8) has a strong synergistic antioxidant action and is one of the core polyphenolic monomers of antioxidants, which is a common component of MA and CI (Zhou, 2012; Cai et al., 2014; Xia et al., 2016a). Therefore, rosmarinic acid 6) and chlorogenic acid 8) became the quality markers for XSJ. Moreover, caffeic acid 5) is the synthetic precursor of these phenolic acids polymers (Chen et al., 2018).

#### 3.1.3 Flavonoids

Flavonoids are an important class of plant secondary metabolites, with 2-phenylchromone as the parent nucleus. It is another major component of active ingredients in XSJ, and all three herbs have high total flavonoid content. 17 flavonoids identified are shown in (Supplementary Figure S3C) and were chiefly divided into flavones and flavonols. Among them, linarin 25) is the characteristic chemical component of CI, which is the quality marker in the Ch.P (Lin et al., 2013). Also, the Ch.P includes rutin 21) as a quality maker of MA (Sun et al., 2016).

#### 3.1.4 Other Constituents

In addition to triterpenes, phenolic acids, and flavonoids, sterols identified in XSJ include β-sitosterol 28) and β-daucosterol 29) (Zhou et al., 2012) (Supplementary Figure S3D). XSJ also contains long-chain fatty acids and alcohols with the molecular formula H_3_C-(CH_2_)_30_-COOH (30) and H_3_C-(CH_2_)_32_-CH_2_OH 31). The long-chain fatty acids were mainly obtained by NMR analysis (Zhou et al., 2012). XSJ is rich in amino acids, and 12 characteristic amino acids were discovered. Four of them are essential amino acids, namely valine (Val), isoleucine (Ile), leucine (Leu), and phenylalanine (Phe), reflecting the TCM’s view of “nourishing yin for lowering fire 养阴扶正以降火,” which is in line with the efficacy of XSJ (Ke et al., 2007). The non-essential amino acids include aspartic acid (Asp), serine (Ser), proline (Pro), etc. The active ingredients in the XSJ herbs are complex and diverse, which still requires systematic active ingredient testing. The results of the phytochemical analysis can assist in confirming the exact pharmacological effects of each active ingredient.

## 4 Biological Activities

In TCM theory and experience, XSJ has the effect of clearing heat and detoxifying toxins and is mainly used to treat wind-heat colds, fever, and sore throat. It is suitable for treating red eyes and headache, hypertension, dizziness and tinnitus, sore throat, furuncles, and swellings caused by wind-heat colds (Chinese Pharmacopoeia Commission, 2020). Because of its mild and long-lasting effect, and no side effects such as “drowsiness and weakness” of chemical medicines for cold and flu. With the rapid development of modern pharmacology and biotechnology, the combination of modern pharmacology and phytochemistry has been used to verify the traditional efficacy of XSJ and explain its mechanisms. Modern research has shown that XSJ granule can inhibit the growth and reproduction of various bacteria, and have stronger inhibitory effects on *Staphylococcus aureus* and *hemolytic streptococcus* (Guo, 2010), indicating that the antipyretic and anti-infective effects are closely related to their antibacterial action. There is growing evidence that the active ingredients in XSJ have pleiotropic effects on tumors, lipid, and glucose disorders (diabetes), bacterial infections, immune system disorders, and liver disease (Huang et al., 2007; Ma et al., 2011; Qiu et al., 2011; Yu et al., 2011) (Supplementary Table S2).

### 4.1 Antiviral Effect

Chinese medicines have unique efficacy in the treatment of influenza (Huang et al., 2021). Extracts of XSJ have significant antiviral effects and are commonly used to treat fever, which was granted a quasi-brand name in 1985 and has been commercially available for nearly 40 years (Guangzhou Xingqun Pharmaceutical, 2022). *In vitro* experiments have shown that the lipid-soluble components of XSJ extract can inhibit influenza A H3N2, H5N1, and B viruses co-cultured with Madin-Darby canine kidney (MDCK) cells at a dose 20 times lower than the cytotoxic dose (Zhan and Dong, 2009). XSJ was found to inhibit the proliferation of respiratory syncytial virus (RSV) and to be protective against RSV-infected rats. Its effective concentration was 544.59 μg/ml, and the efficacy was enhanced with an increasing dose of the drug, which also reduced the virus titer in tissues and prevented virus replication *in vivo*, and the anti-RSV effect was similar to that of the same dose of virazole (Huang et al., 2007). Viruses with 1:256∼1:2048 dilution of XSJ infusion and 1:128 dilution of XSJ granule have a cytopathogenic effect on Coxsachie (Cox) A16 *in vitro* at the cellular level (Yao et al., 2017b). Meanwhile, XSJ granule produces significant anti-dengue virus (DENV)-1 effects by *in vitro* pre-administration, which has a similar effect to ribavirin injection. MTT results showed that the maximum nontoxic dose of XSJ granule on the C6/36 cells was 7.81 mg/ml. The cytopathic effect of DENV-1 infection was measured after treatment with XSJ granule solution, and the survival rate of cells infected with the virus increased, which significantly attenuated the effect of virus-induced cytopathy. Cellular immunofluorescence assay showed that the relative infection rate of DENV-1 was decreased after pretreatment with different dilutions of XSJ granule. The inhibition of the virus copy number by XSJ granule was obvious. It is suggested that XSJ directly affects DENV-1, probably by direct inhibition of viral infectivity and RNA replication capacity (Yao et al., 2017a; Zhang et al., 2019a).

A study further investigated the effect of XSJ on influenza A H1N1 virus and its mechanism at the molecular level. By observing the effect of XSJ on the replication process of MDCK cells, the half toxic concentration (TC50), half infectious amount (TCID50), and half inhibitory concentration (IC50) were calculated. The action of XSJ on influenza A H1N1 virus inhibited vacancy formation, nuclear export of viral nucleoproteins (NPs) and phosphorylation of nuclear factor kappa-B (NF-κB) pathway-related proteins Iκκα, Iκκβ, NF-κB p50, and NF-κB p65 in the virus strain. XSJ has a significant inhibitory effect on influenza A H1N1 virus, probably through the inhibition of NF-κB pathway-associated protein phosphorylation (Yu et al., 2018).

Aqueous extracts of natural herb PV can interrupt SARS-CoV-2-Spike glycoprotein binding to its receptor angiotensin-converting enzyme 2 (ACE2) and block the viral entry step (Ao et al., 2021). Aqueous extracts of PV also inhibited the HIV-1 (Oh et al., 2011), IHNV (Li et al., 2019), Ebola virus (Zhang et al., 2016), and HSV-1 (Nolkemper et al., 2006) by early interference. The polysaccharide and essential oils from CI inhibited replication of anti-duck hepatitis A virus (Ming et al., 2017; Ming et al., 2019), HSV-1, HAV, and VSV (Youssef et al., 2020). Caffeic acid and chlorogenic acid from aqueous and hydro methanolic extracts of MA inhibited the replication of human CoV and single-stranded RNA viral strains (Thabti et al., 2020). Caffeic acid, the active ingredient of the three herbs of XSJ, can inhibit Hepatitis C virus (HCV) propagation and the proliferation of influenza A virus by interfering mainly with the viral genome replication in the infected cells (Tanida et al., 2015). Caffeic acid has also been shown to have antiviral activity against the herpes simplex virus (DNA virus) and polio virus (RNA virus) (Utsunomiya et al., 2014). The inhibition effect indicated that phenolic acids, like rosmarinic acid (Nolkemper et al., 2006), caffeic acid (Nolkemper et al., 2006; Thabti et al., 2020), ursolic acid (Li et al., 2019), chlorogenic acid (Thabti et al., 2020), were the active ingredients of antivirus.

### 4.2 Antioxidant Activity

Antioxidant herbs mainly belong to bitter-cold herbs, while oxidizing herbs are mainly warm (Liao et al., 2008). The extract of XSJ contains phenolic acids, flavonoids, polysaccharides, and amino acids, which have the effects of lowering blood pressure, dilating coronary arteries and preventing coronary atherosclerosis, etc. It is a kind of natural organic antioxidant. A study was carried out to compare the scavenging effect of different systems on free radicals in terms of absorbance size by extracting XSJ with ethanol, followed by petroleum ether, ethyl acetate, and n-butanol in turn (Ma et al., 2011). The results showed that all extracted fractions of XSJ had some scavenging effect on hydroxyl radicals, except for the petroleum ether layer. Compounds with catechol functional groups, such as caffeic acid and its derivatives, including rosmarinic acid, and flavonoids, such as rutin, which are active substances for scavenging reactive free radicals, have been reported in extracts of XSJ (Ma et al., 2011). The antioxidant activity of active ingredients was conferred by the structural property of phenolic compounds, which can directly scavenge of reactive oxygen species (ROS) or metal chelation (Habtemariam, 2019).

Two ursane-type triterpenes from ethanol extract of PV induced heme oxygenase-1(HO-1) in HepG2 cells (Jeong et al., 2008) and ursolic acid inhibited nitric oxide (NO) (Ryu et al., 2000; Miceli et al., 2005). Caffeic acid, rosmarinic acid, rutin, quercetin, and luteolin from the total phenols of PV extract increased superoxide dismutase (SOD) activity and decreased malondialdehyde (MDA) content in the serum of tumor-bearing mice (Feng et al., 2010). 1,1-diphenyl-2-picryhydrazyl (DPPH) and 2,2′-Azinobis-(3-ethylbenzthiazoline-6-sulphonate) (ABTS) free radicals were significantly scavenged by the ethanol extracts of PV, which is mainly determined by ursolic acid, oleanolic acid, rosmarinic acid, salviaflaside, caffeic acid, and hyperoside (Chen et al., 2019). Rosmarinic acid and caffeic acid from PV reduced breakage together with the apoptotic process, eliminated ROS production, and diminished IL-6 release (Vostalova et al., 2010), and induced the expression of efflux transporters through activation of the Nrf2-mediated signaling pathway (Wu et al., 2016). Chlorogenic acid, rutin, astragalin, quercetin, and kaempferol from MA reduced the ROS and NO production (Kwon et al., 2017), and scavenged DPPH radical (Katsube et al., 2009). Total phenolic and flavonoid from CI showed the peroxyl radical-scavenging capacity, and quercetin, luteolin, acacetin, luteolin 7-O-glucoside, linarin, luteolin, chlorogenic acid, apigenin also exhibit such activity (Luyen et al., 2015; Hwang et al., 2016; Kang et al., 2021). As the quality marker of XSJ, rosmarinic acid can scavenge ROS, inhibit lipid peroxidation (Elufioye and Habtemariam, 2019) and activate the AMPK pathway (Feng et al., 2020). And linarin activated Nrf-2 through activating PI3K/Akt signaling pathway in myocardial cell H9C2 and langendorff heart model (Yu et al., 2017). In addition, the aqueous extract of PV may mediate the protective effect of gastric ulcers by inhibiting oxidative stress and promoting regenerative processes in the mucosa. Considerable amounts of hyperoside, rutin, isoquercitrin, small amounts of kaempferol, apigenin, and phenolic acids such as caffeic acid are present in the aqueous extract (Grosan et al., 2020a). The higher the level of total phenolic content and rosmarinic acid content, the stronger the total antioxidant capacity of PV (Sarosi et al., 2011) (Supplementary Figure S4).

### 4.3 Anticancer Activity

The ancient Chinese medical treatise “Zhongzangjing” describes cancer-like symptoms such as “Yong, Yang, Shang, and Zhong,” caused by the retention of various pathogens such as heat and dampness. Tumor growth involves the induction of cell cycle processes, avoidance of apoptosis and activation of cell survival pathways. Studies have shown that some of the active ingredients in XSJ can inhibit the proliferation of cancer cells *in vitro* and suppress tumor growth *in vivo* (Yu et al., 2011).

PV extract inhibits the growth of proximal tubular epithelial cells (PTC) *in vivo* and *in vitro via* autophagy, which is associated with the AMPK/mTOR/ULK1 pathway (Song et al., 2021a). PV extract also inhibits the proliferation and migration of thyroid cancer cells both *in vitro* and *in vivo*, which may have been achieved by modulation of the expression of MKI67, PCNA, and CDH1 (Yu et al., 2021). PV total flavonoids have an obvious anti-hepatocarcinoma effect, and the mechanism may be linked to the inhibition of autophagy and promotion of apoptosis in liver cancer cells, which may be related to the activation of the PI3K/Akt/mTOR pathway (Song et al., 2021b). Root extract of PV shows the anticancer by suppressing angiogenesis, inducing apoptosis and cell cycle arrest, whose mechanism may be *via* activating PI3K/AKT signaling pathway (Gao and Xu, 2019). It was also associated with the enhancement of Bax expression and the decrease in the expression of Bcl-2. Ethanol extract of PV activated p53-mediated apoptosis (Kim et al., 2020) and the proapoptotic protein caspase-3 induced cellular apoptotic pathway (Zhu et al., 2018). And the CI Extract induces apoptosis by suppressing constitutive STAT3 activation in human prostate cancer DU145 cells, especially the methylene chloride fraction acacetin of CI could inhibit the JAK1/2 and STAT3 signaling pathways (Kim et al., 2013). Linarin from CI induced factor-related apoptosis-induced ligand (TRAIL)-regulated apoptosis *via* upregulating Caspases (Xu et al., 2017) and p53 expression, and inhibiting NF-κB/p65 expression (Zhen et al., 2017). Total triterpenes and total phenols from 95% ethanol extract of PV increased the TNF-α level, such as caffeic acid, rosmarinic acid, rutin, quercetin, oleanolic acid, and ursolic acid (Feng et al., 2010; Lee et al., 2019; Zheng et al., 2022). Chlorogenic acid, rutin, quercetin, astragalin, and kaempferol from MA also recovered the effects of endoplasmic reticulum stress-induced resistance to DOX through COX-2 or p38 MAPK-mediated inactivation of the PI3K/Akt pathway (Yang et al., 2020). The activity of luteolin and acacetin-7-O-rutinoside from water extract of CI was confirmed to be similar to that of tyrosinase inhibitor (Choi et al., 2016) (Supplementary Figure S5).

In the study results, considering the dose and drug delivery of oral granule, anticancer studies of XSJ are not systematic, so no antitumor effect of XSJ granule has been reported. But these three herbs were shown to inhibit tumor growth in a dose-dependent manner, respectively. Oleanolic acid, ursolic acid, linarin, and other flavonoids may be active compounds showing targeted antitumor activity. Further research on anti-tumor treatment of the whole XSJ formula should be carried out. The emergence of severe side-effects and multidrug resistance (MDR) are big challenges to clinical usage of cancer chemotherapy. Therefore, the synergistic treatment of XSJ reflects its advantages, such as reducing the toxic and side effects of chemotherapy, so it could serve as a putative adjuvant drug with chemotherapy in the future (Yang et al., 2011; Yang et al., 2017).

### 4.4 Antidiabetic Activity

Diabetes mellitus belongs to the category of “thirst (Xiao Ke)” in Chinese medicine, and the patient may suffer from physical wasting, excessive urination, excessive drinking, excessive eating, and sweet-tasting urine. Aqueous extract of PV attenuated IL-1β-increased NF-κB binding activity and inflammatory cytokine expression (Wu et al., 2012), and disrupted the TGF-β/Smad signaling (Namgung et al., 2017). Rosmarinic acid, caffeic acid, rutin, and quercetin form PV increased serum-insulin, attenuation of α-amylase and α-glucosidase (Raafat et al., 2016). Aqueous extract of MA improved glucose metabolism disorders, ameliorated the antioxidative ability, and decreased insulin resistance *via* IRS-1/PI3K/Glut-4 signaling pathway (Cai et al., 2016; Lyu et al., 2021). Both MA extract (Wu et al., 2017) and CI water extract (Nepali et al., 2018) exert anti-diabetic activity by regulating the AMPK pathway in diet-induced obesity mice models. Chlorogenic acid, caffeic acid, rutin, isoquercitrin astragalin, and dicaffeoylquinic acid from MA improved glucose tolerance and lowered the level of glucose (Ma et al., 2016). Acacetin, apigenin, chlorogenic acid, kaempferol, luteolin, and quercetin from CI improved the fat metabolism (Bai et al., 2018) and inhibited the formation of advanced glycation end products (Tsuji-Naito et al., 2009). Triterpenes acid from PV controlled blood glucose and decreased SOD mRNA expression in pancreatic *β* cells (Zhou et al., 2013). Total polyphenols, total flavonoid aglycons, quercetin, and kaempferol from MA exhibited a hypocholesterolemic and hypotriacylglyceridemic effects attributed to the stimulatory effect on the β-oxidation of fatty acids. They showed the induction of fatty acid oxidation, inhibition of lipogenesis, and suppression of oxidative stress (Kobayashi et al., 2010). Among them, chlorogenic acid, rutin, quercetin derivatives and β-sitosterol from MA also showed the significant control of lipid and glucose metabolism, attenuation of oxidative stress and insulin resistance improvement (Hunyadi et al., 2012; Sun et al., 2015; Sheng et al., 2018). β-Sitosterol can activate the synthesis and translocation of the transporter GLUT-4 (Ponnulakshmi et al., 2019). In summary, some of the active ingredients of XSJ can improve insulin resistance, increase the sensitivity of surrounding tissues to insulin, and enhance the ability of fat cells to take up and utilize glucose, resulting in lower blood glucose (Supplementary Figure S6). It is important to conduct studies with diabetic patients and healthy volunteers to understand how natural products such as MA work in the human body. A double-blind clinical trial reported that 12 weeks of MA extract in patients with type 2 diabetes reduced malondialdehyde (MDA) levels but did not affect other biomarkers of inflammation and oxidative stress (Taghizadeh et al., 2022). Consumption of MA extract for 12 weeks significantly reduced triglyceride, VLDL-cholesterol, and MDA levels, and significantly increased HDL-cholesterol and GSH concentrations in T2DM patients with kidney disease (Taghizadeh et al., 2017). To evaluate the effects of DNJ-rich MA extract on patients with T2D, the results of a clinical trial combining dietary control, exercise and the total alkaloid composition of MA showed that it lowered blood glucose, regulated blood lipids and had fewer side effects compared to acarbose treatment (Liu et al., 2013). However, studies on safety are lacking and studies on the absorption, distribution, metabolism and excretion of compounds in XSJ need to be conducted.

### 4.5 Antibacterial Activity

Antibacterial herbal medicines are mostly heat-clearing and detoxifying drugs, mostly with anti-microbial and anti-inflammatory effects. XSJ has an antibacterial effect on *Staphylococcus aureus* and *Streptococcus hemolyticus* (Guo, 2010). XSJ extract inhibits *Escherichia coli* (*E. coli*) in human urine samples, and aqueous extract showed higher inhibitory activity than ethanol extract (Komal et al., 2018). The active ingredients chlorogenic acid, caffeic acid, protocatechuic acid, luteolin-7-glucoside, apigenin-7-glucoside, kaempferol-3-glucoside, linarin, apigenin, luteolin, and kaempferol have been observed in CI extract studies to have an inhibition of bacteria activity (Kozyra et al., 2015). CI extract can inhibit gram-positive bacteria, such as *Streptococcus aureus* and *Streptococcus pneumoniae*, as well as *Streptococcus epidermidis*, *Bacillus cereus* and *Bacillus subtilis*. The 50% and 80% methanol extracts and fractions are richer in phenolic compounds that showed antibacterial activity (Kozyra et al., 2015). Silver nanoparticles of CI aqueous extract inhibited *Klebsiella pneumonia*, *E. coli*, and *Pseudomonas aeruginosa*, which contained total flavonoids, terpenoids, and glycosides (Arokiyaraj et al., 2014; Arokiyaraj et al., 2015). Rosmarinic acid and caffeic acid from PV inhibited Gram-positive bacteria (Psotova et al., 2003), while oleanolic acid and ursolic acid from MA inhibited periodontogenic bacteria (Park et al., 2014). The aqueous and hydroalcoholic extracts of PV showed antibacterial effect against most bacteria, showing measurable activity against Gram-positive bacteria and multi-drug resistant Gram-negative bacteria. It indicates that the extract of PV has antibacterial potential in the adjuvant treatment of multi-drug resistant infections (Grosan et al., 2020b).

Ursolic acid compromised the integrity of the bacterial membrane, inhibited protein synthesis, and elicited the oxidative response in Methicillin-Resistant *Staphylococcus aureus* (MRSA) *via* the AhpC induction (Wang et al., 2016). Caffeic acid could inhibit bacterial enzyme activity, including respiratory enzymes against the representative foodborne bacteria *E. coli* O157:147, *Salmonella Typhimurium*, and *Listeria monocytogenes* (Park and Kang, 2021). Chlorogenic acid induces damage of intracelluar and outer membranes as well as disruption of cell metabolism resulting in *Salmonella Enteritidis* S1 death eventually. It also suppressed the activities of malate dehydrogenase and succinate dehydrogenase, two main metabolic enzymes in the TCA cycle and electron transport chain (Sun et al., 2020). β-Sitosterol showed antibacterial activity against *Staphylococcus aureus* and *E. coli* (Ododo et al., 2016). Since ancient times, these three herbs and XSJ have been used for their antibacterial and anti-inflammatory properties. A randomized clinical trial of chronic infective refractory wounds repair focused on the decoction of PV and showed higher rates of wound repair, bacterial negativity, clinical total efficacy rate and shorter healing times compared to routine wound dressing change. It inhibited the growth of traumatic bacteria and provides a good environment for the growth of granulation. Compared with modern drugs, PV does not produce too many toxic side effects, which is of some relevance especially in the case of antibiotic abuse (Zhao et al., 2021).

### 4.6 Immunomodulatory Effect

The active ingredient rosmarinic acid in XSJ and aqueous extract of PV inhibited Th1, Th2, and Th17 immune responses *via* inhibiting HMGB1/TLR9 signaling (Guo et al., 2021; Zhu et al., 2022).Triterpenoids, flavonoids, tannins and polysaccharide from PV suppressed Con A-, LPS-, and OVA-induced splenocyte proliferation in the immunized mice, reduced total IgG, IgG1 ,and IgG2b levels significantly, and suppressed the cellular and humoral response (Sun et al., 2005), immuno-stimulating activities through the activation of TLR2, TLR4 ,and CR3 (Li et al., 2015). Ursolic acid, quercetin, β-sitosterol from PV suppressed the activation of NF-κB and interferon regulatory factor 3 (IRF3), and inhibited genes related to antigen presentation pathways (Chen et al., 2020a). Water extract of MA stimulated the production of NO and PGE2 as immune response parameters, and was associated with the increased expression of inducible NO synthase and COX-2 (Kwon et al., 2016). Extract of CI increased the delayed-type hypersensitivity (DTH) reaction, and enhanced antibody generation and IgG and IgM levels in mice sera (Cheng et al., 2005). β-sitosterol in XSJ regulates immunity by increasing viable peripheral blood mononuclear cell (PBMC) numbers and it activates swine dendritic cells (DCs) in culture. It can drive the BMDC response towards a Th1 pattern with IFN-α secretion and the absence of IL-10 (Fraile et al., 2012). Therefore, XSJ has a potential immunomodulatory effect.

### 4.7 Hepatoprotective Activity

The liver is essential for bile formation, amino acid utilization and ammonia detoxification. In TCM, the liver is an organ susceptible to heat and toxins, so its detoxification capacity is diminished by pathological damage. XSJ is rich in bioactive flavonoids and polyphenols, which restore the balance of the liver’s metabolic disorder state. Flavonoids and total phenolic extracts obtained from floral spikes of PV have hepatoprotective potential and free radical scavenging *in vivo* (Ahmad et al., 2020). 80% methanol extract of PV reduced the contents of inflammatory factors and liver function markers, and improved metabolic disorder of liver injury (Deng et al., 2021). Extract from CI reduced the elevated levels of ALT and AST, alleviated abnormal alterations in structure and function and liver (Zhang et al., 2019b), and inhibited bioactivation of hepatotoxicity and downregulated CYP2E1 expression (Jeong et al., 2013). The active ingredient rosmarinic acid scavenged or reduced reactive superoxide or peroxynitrite, decreased indicators of hepatotoxicity, inhibited hepatic stellate cell proliferation, suppressed the activities of TGF-β1, CTGF, and α-SMA, attenuated fibrosis, improved biochemical indicators and histopathological patterns (Osakabe et al., 2002; Rocha et al., 2015; Elufioye and Habtemariam, 2019). β-Sitosterol, rutin, and isoquercitrin from MA extract reduced the production of NO, malondialdehyde, and glutathione levels, and prevented the increase in the hepatic malondialdehyde, playing a pivotal role in the antifibrotic properties (Amer et al., 2013). β-sitosterol downregulated the expression of apoptosis-related genes in the PI3K/Akt pathway and restored the liver enzymes, liver lipid peroxidation markers, total bilirubin, and albumin to their normal levels without inhibitory effect on the CYP2E1 activity (Abdou et al., 2019; Chen et al., 2020b). Apigenin-7-glucoside suppresses the elevation of GPT, GOT, MDA, and 8-OHdG, and inhibits the reduction of GSH in a dose-dependent manner *in vivo* and reduces the damage of hepatocytes *in vitro* (Zheng et al., 2005). The low dosage of oleanolic acid reprogrammed the liver to activate the Nrf2 pathway in mice (Liu, 1995; Jin et al., 2012; Liu et al., 2019). In general, the regulatory and protective effects of XSJ on the liver are caused by its specific active ingredients (Supplementary Figure S7).

## 5 Quality Control

Research on quality standards of proprietary Chinese medicines is generally based on thin-layer chromatography (TLC) and HPLC methods. The quality control study of XSJ focuses on applying HPLC methods for the content determination of various components and the establishment of fingerprint profiles. The Drug Standard of the Ministry of Health of the People’s Republic of China, Book XV, shows that the quality control of XSJ includes the identification reaction of chloroform solution saturated with antimony trichloride and the TLC identification of ursolic acid, excluding the content of XSJ fingerprinting and content dete8mination. However, the methods of compound identification or content determination for individual active ingredients can hardly reflect the quality of XSJ comprehensively. To provide a reference for the quality standard of the XSJ granule, most of the studies reported that the fingerprinting of XSJ extracts as a whole had been carried out, focusing on the development and determination of several main active ingredients. Ch.P (2015 and 2020) summarized and extracted the previous studies, mainly based on TLC identification, particle examination, fingerprinting and content determination as indicators for the determination of XSJ granule. The authenticity of XSJ granule was controlled. The quality markers of XSJ granule included in the Ch.P are chlorogenic acid, rosmarinic acid and linarin, and the content determination is calculated by the amount of PV in each bag with rosmarinic acid content. The three quality markers were significantly show in the fingerprint of Xiasangju, and it requires that the similarity between the fingerprint of the test sample and the control fingerprint should not be less than 0.90. The content of rosmarinic acid in PV is determined to be not less than 2.5 mg/bag (Chinese Pharmacopoeia Commission, 2020).

### 5.1 Quality Standards

With the development of various identification techniques, the researchers identified the authenticity of XSJ granule by HPLC and UPLC. The fingerprint profile of the extract was analyzed as a whole and focused on the sequence and interrelationship of each constituent fingerprint peak. It is important as a quality control method in optimizing the production process. Ke et al. (2008) established fingerprint profiles of 10 batches of XSJ granule with similarity greater than 0.970, and compared the fingerprint profiles of the aqueous decoction, alcoholic precipitation, concentrated solution, and finished products of the herbs, respectively, and found that there were significant differences. Based on establishing the fingerprint profiles of amino acid components (Ke et al., 2007) and alcohol extracts of XSJ, the fingerprint profiles of different brands of XSJ granule were compared, and qualitative and semi-quantitative evaluations were conducted using similarity analysis and cluster analysis (Yao et al., 2012). The control chart comparison method is simple and intuitive, fully reflecting the overall characteristics of XSJ granule and effectively controlling the product quality. Xia et al. (2014) constructed the fingerprint profiles of 12 batches of XSJ granule by UPLC and labelled 16 common peaks with fingerprint similarity above 0.9. HPLC was applied to establish the quality standards of different dosage forms of XSJ granule. The fingerprint profiles of sugar- and sugar-free XSJ granule were analyzed by Principal Components Analysis (PCA) and Partial Least Squares Discriminant Analysis (PLS-DA), in which the three main components, salviaflaside, luteolin 7-O-glucoside and linarin, were found to be different (Xia et al., 2016b). And this quality difference may come from the ethanol extraction of CI alone, which facilitates the exudation of its flavonoid fractions. Also, these three compounds are among the most reported substances in the literature as quality control markers. Further, the random forest algorithm was used to process similar samples of XSJ with better results than the PCA and PLS-DA algorithms (Xia et al., 2017). A study on the change of the compound composition of XSJ detected the production of new unknown chemical components and high content of the three herbs after decoction together, and the content of some major chemical components also changed (Peng et al., 2014). The single herb preparation and the negative preparation (missing PV, MA, and CI, respectively) were examined under the same conditions. The HPLC fingerprints showed that six characteristic peaks originated from PV, one from MA, five from CI, one from MA, and CI together, and one from PV, MA, and CI together, and the peak areas of these characteristic peaks changed to different degrees before and after the compatibility (Xia et al., 2016a).

### 5.2 Content Determination

Quantitative analysis of XSJ focused on the determination of rosmarinic acid (Lin et al., 2012a; Huang, 2013; Lin et al., 2013; Peng et al., 2014; Zhou et al., 2014; Luo et al., 2016; Sun et al., 2016), salviaflaside (Hua et al., 2013; Lin et al., 2013), chlorogenic acid (Lin et al., 2013; Sun et al., 2016), linarin (Zeng and Ding, 2008; Lin et al., 2012a; Lin et al., 2013), luteolin 7-O-glucoside (Sun et al., 2016), rutin (Sun et al., 2016), quercetin (Sun et al., 2016), and total saponins (Wu, 2019). Most samples had the highest contents of chlorogenic acid and rosmarinic acid, followed by salviaflaside, and the other components varied widely among batches of the herbs.

A method was established for the quantitative determination of rosmarinic acid and linarin in XSJ granule by HPLC. 15 batches of samples were extracted by methanol ultrasonication and analyzed within 15 min. The results showed that the amounts of rosmarinic acid and linarin in the measured samples varied considerably, and the content determined to be not less than 1.50 and 0.50 mg/bag (Lin et al., 2012a). HPLC was used to simultaneously determine the contents of rosmarinic acid and salviaflaside in XSJ granule. Using acetonitrile−0.1%-phosphoric acid gradient elution, rosmarinic acid, and salviaflaside peak shape are good, and the separation is ideal, negative without interference, the content of rosmarinic acid is not less than 0.22 mg/g (Hua et al., 2013). Further study determined the contents of chlorogenic acid, salviaflaside, rosmarinic acid, and linarin in XSJ granule simultaneously by Reversed-phase HPLC method, and compared the contents of active ingredients from different manufacturers. The result suggested that the lower limits of the contents of the four active ingredients in the quality standard were 1.50, 0.20, 2.00, and 1.00 mg/bag for chlorogenic acid, salviaflaside, rosmarinic acid, and linarin, respectively (Lin et al., 2013). Based on the study of XSJ granule, quality control of other preparations of XSJ has also been reported in the literature. For example, the quality research of XSJ herbal tea mainly focuses on establishing quality control methods for chlorogenic acid, rosmarinic acid, luteolin 7-O-glucoside, rutin, quercetin and linarin, and contents determination by HPLC (Sun et al., 2016). Since chlorogenic acid is a common component of all three herbs, it is difficult to be used as a quantitative standard. Rosmarinic acid in PV was used for content determination for quantification.

## 6 Conclusion

After years of practice, XSJ has been practically used to treat influenza and purge heat and toxicity. It is sold in several regions and occupies a certain market share for its good quality. The latest studies of XSJ focus on the development of active ingredients, reflecting the active characteristics of triterpenes, phenolic acids, and flavonoids. XSJ may benefit people with liver disease by preventing or treating liver injury, and may have one or more of antioxidant, antifibrotic, immunomodulatory, or antiviral activities. However, “liver-protective” herbs may also cause liver damage, so it may have to take into account the hepatotoxicity of XSJ. For example, whereas a low dose of oleanolic acid is hepatoprotective, higher doses, and long-term use of oleanolic acid can produce liver injury, characterized by cholestasis. This paradoxical hepatotoxic effect occurs not only for oleanolic acid, but also for other oleanolic acid-type triterpenoids. Dose and length of time of oleanolic acid exposure differentiate the ability of acid to cause hepatoprotection or hepatotoxicity (Liu et al., 2019). And because of the incomplete study of XSJ phytochemical composition, more information can be obtained only from the study of the three herbs.

In herbal formulas, these fundamental problems or phenomena have never been completely resolved. The mismatch of chemical composition and active ingredients has resulted in potent substances that remain unclear, but effective for clinical use. The boundary between pharmacological and toxic effects is unclear, leading to unclear synergistic or antagonistic mechanisms. The *in vivo* delivery process of herbal medicinal substances is not clarified. Therefore, more phytochemical composition, physical structure composed of multiple components, and biological activities of XSJ should be addressed, and further application of advanced technical tools, such as network pharmacology and structural Chinese medicine, to systematically study the complete pharmacological substance basis of the compound. The synergistic treatment of XSJ could be a potential putative adjuvant drug with chemotherapy in the future.
