# Supplementary material for: Phytochemistry, Pharmacology and Quality Control of Xiasangju: A Traditional Chinese Medicine Formula
Source: Front Pharmacol. 2022 Jun 23;13:930813. doi: 10.3389/fphar.2022.930813 (PMC9259862; doi:10.3389/fphar.2022.930813)
Supplement: Supplementary file 1 [file DataSheet1.docx]

Supplementary Material

# Supplementary Figures and Tables


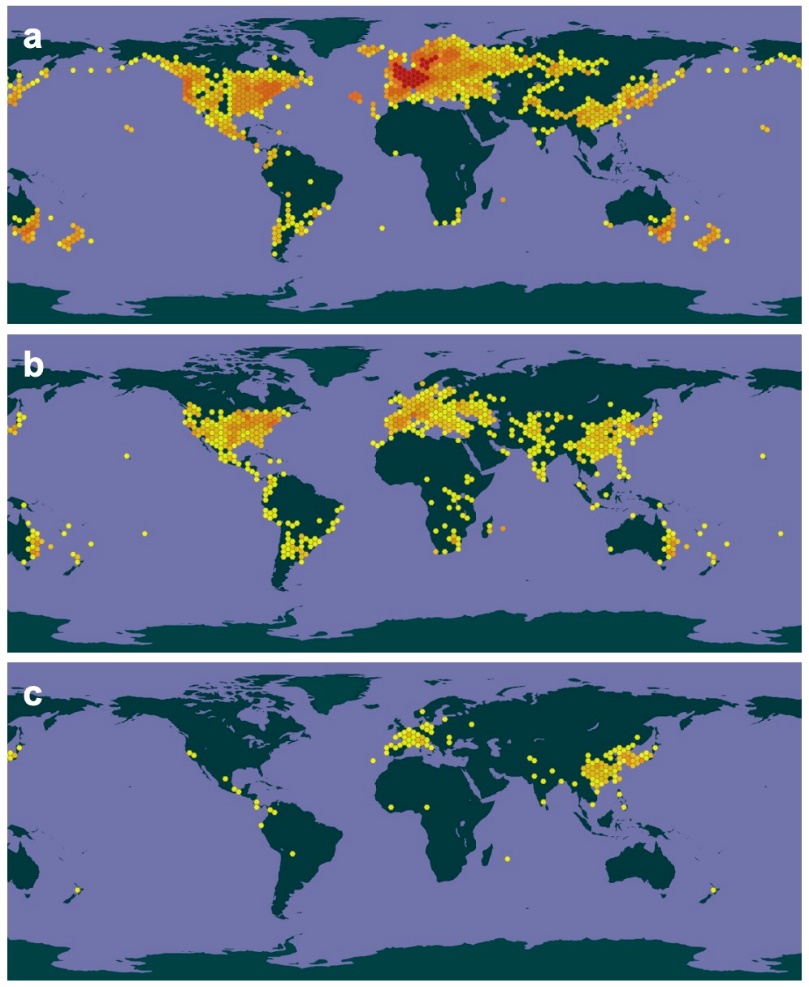


**Figure 1.** Geographical distribution of a) *Prunella vulgaris* L., b) *Morus alba* L, and c) *Chrysanthemum indicum* L. Data from the *Global Biodiversity Information Facility.*


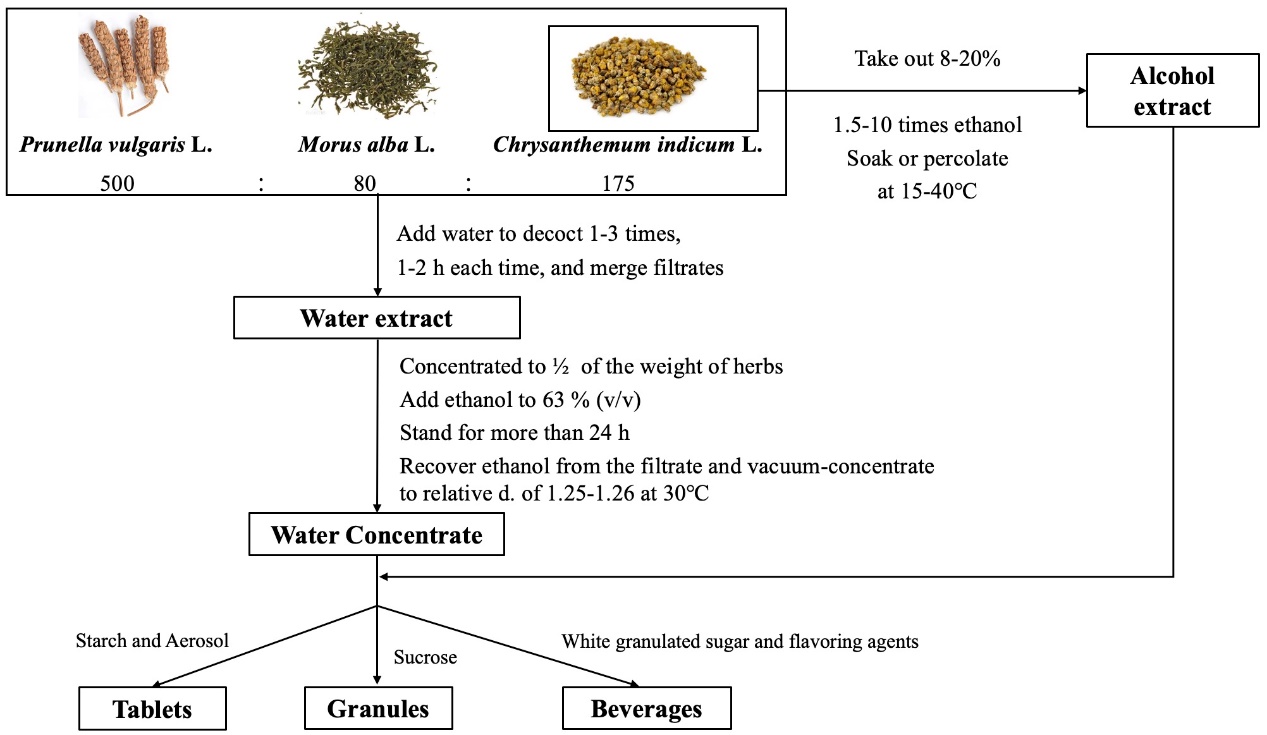


**Figure 2.** Extraction and processing technology of XSJ formulation.

**Table 1 Phytochemical composition of XSJ**

| NO. | Chemical compound | Molecular formula | Type | Source | References |
| --- | --- | --- | --- | --- | --- |
| 1 | Oleanolic acid | C_30_H_48_O_3_ | Triterpenes | a | (Wu, 2019) |
| 2 | β-Amirin | C_30_H_50_O | Triterpenes | a | (Zhou et al., 2012; Zhou, 2012) |
| 3 | Ursolic acid | C_30_H_48_O_3_ | Triterpenes | a | (Zhou et al., 2012; Zhou, 2012) |
| 4 | Protocatechuic acid | C_7_H_6_O_4_ | Phenolic acids | a, b | (Xin and Tang, 2013) |
| 5 | Caffeic acid | C₉H₈O₄ | Phenolic acids | a, b, c | (Xin and Tang, 2013) |
| 6 | Rosmarinic acid | C_18_H_16_O_8_ | Phenolic acids | a | (Hua et al., 2013; Xia et al., 2016a) |
| 7 | Salviaflaside | C_24_H_26_O_13_ | Phenolic acids | a | (Hua et al., 2013; Xia et al., 2016a) |
| 8 | 1-Chlorogenic acid | C_16_H_18_O_9_ | Phenolic acids | b, c | (Lin et al., 2013; Zhou, 2012; Xia et al., 2016a; Cai et al., 2014) |
| 9 | Caffeoylquinic acid | C_16_H_18_O_9_ | Caffeic acid derivative | b | (Xin and Tang, 2013) |
| 10 | Dicaffeoylquinic acid | C_25_H_24_O_12_ | Caffeic acid derivative | b, c | (Xin and Tang, 2013) |
| 11 | Kaempferol | C_15_H_10_O_6_ | Flavonoids | a, b, c | (Zhou et al., 2012; Liu et al., 2012; Zhou, 2012) |
| 12 | Quercetin | C_15_H_10_O_7_ | Flavonoids | a, b, c | (Zhou et al., 2012; Liu et al., 2012; Zhou, 2012) |
| 13 | Apigenin | C_15_H_10_O_5_ | Flavonoids | c | (Zhou, 2012) |
| 14 | Luteolin | C_15_H_10_O_6_ | Flavonoids | a, c | (Zhou et al., 2012; Liu et al., 2012; Zhou, 2012) |
| 15 | Acacetin | C_16_H_12_O_5_ | Flavonoids | c | (Zhou, 2012) |
| 16 | Diosmetin | C_16_H_12_O_6_ | Flavonoids | c | (Zhou, 2012) |
| 17 | Astragalin | C_21_H_20_O_11_ | Flavonoids | a, b | (Zhou et al., 2012; Xin and Tang, 2013; Zhou, 2012) |
| 18 | Kaempferol 3-O-rutinoside | C_27_H_30_O_15_ | Flavonoids | a, b | (Xin and Tang, 2013) |
| 19 | Quercetin 3-glucoside | C_21_H_20_O_12_ | Flavonoids | a, b | (Zhou et al., 2012; Xin and Tang, 2013; Liu et al., 2012; Zhou, 2012) |
| 20 | Hyperoside | C_21_H_20_O_12_ | Flavonoids | a, b | (Xin and Tang, 2013) |
| 21 | Rutin | C_27_H_30_O_16_ | Flavonoids | a, b | (Xin and Tang, 2013; Liu et al., 2012; Zhou, 2012) |
| 22 | Apigenin 7-O-glucoside | C_21_H_20_O_10_ | Flavonoids | c | (Xin and Tang, 2013) |
| 23 | Luteolin 7-O-glucoside | C_21_H_20_O_11_ | Flavonoids | c | (Xia et al., 2016b; Xin and Tang, 2013; Liu et al., 2012; Zhou, 2012) |
| 24 | Tilianin | C_22_H_22_O_10_ | Flavonoids | a, c | (Zhou et al., 2012; Xin and Tang, 2013; Zhou, 2012) |
| 25 | Linarin | C_28_H_32_O_14_ | Flavonoids | c | (Zhou et al., 2012; Xin and Tang, 2013; Zhou, 2012; Xia et al., 2016a; Cai et al., 2014) |
| 26 | Acacetin 7-O-β-D-glucuronopyranosyl-(1→2)[α-L-rhamnopyranosyl-(1→6)]-β-D-glucopyranoside | C_34_H_42_O_18_ | Flavonoids | c | (Zhou, 2012) |
| 27 | Diosmetin 7-O-β-D-glucoside | C_22_H_22_O_11_ | Flavonoids | c | (Zhou, 2012) |
| 28 | β- Sitosterol | C_29_H_50_O | Sterols | a, b | (Zhou et al., 2012; Zhou, 2012) |
| 29 | β-Daucosterol | C_35_H_60_O_6_ | Sterol glycoside | b | (Zhou et al., 2012; Zhou, 2012) |
|  | Dotriacontanoic acid | C_32_H_64_O_2_ | Long-chain Fatty Acids | a | (Zhou et al., 2012; Zhou, 2012) |
| 31 | n-nonadecanol | C_34_H_70_O | Others | a | (Zhou et al., 2012; Zhou, 2012) |

a: Prunellae spica (*Prunella vulgaris* L.)

b: Mori folium (*Morus alba* L.)

c: Chrysanthemi indici flos (*Chrysanthemum indicum* L.)


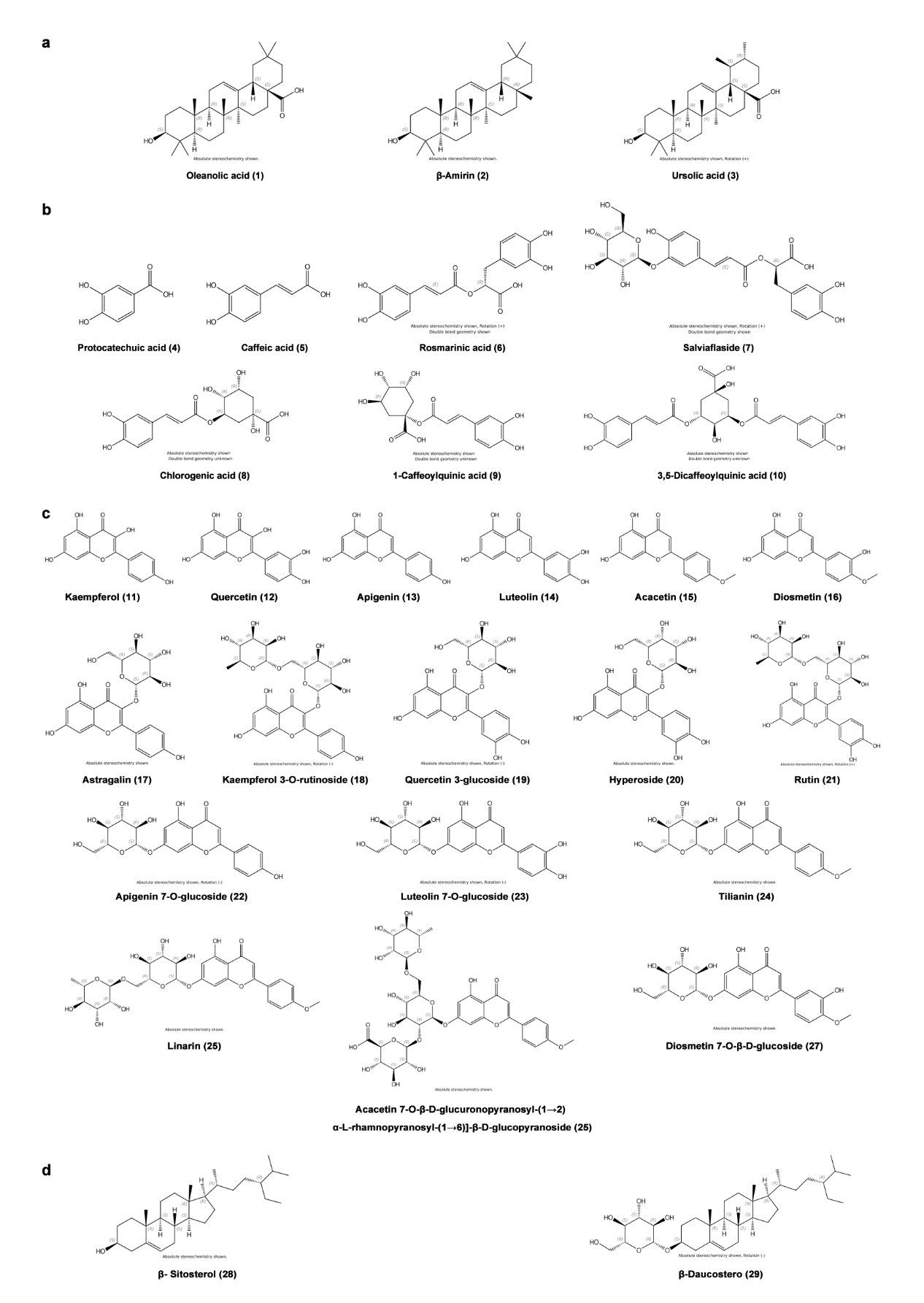


**Figure 3.** Structures of the main constituents isolated from XSJ. a) triterpenes; b) phenolic acids; c) flavonoids; d) sterols.

**Table 2 Pharmacological activity and mechanisms of XSJ**

| Biological Effect | Target or Possible Mechanism | Models | Active Ingredient or Marketed Drug | References |
| --- | --- | --- | --- | --- |
| Antiviral effects | - | Influenza A virus subtype H3N2  Influenza A virus subtype H5N1  Influenza B virus strains  Madin-darby canine kidney cells line (MDCK) | XSJ herbal crude powder aqueous extracts | (Zhan and Dong, 2009) |
|  | - | Respiratory syncytial virus strain (RSV)  Human Epithelioma-2 (Hep-2) cells  BALB/c mice | XSJ granule concentrate | (Huang et al., 2007) |
|  | - | Coxsackievirus 16 (CA16)  Vero cells | XSJ herbal crude infusion  XSJ granules | (Yao et al., 2017b) |
|  | Inhibited the plaque formation, the export of nucleoprotein (NP) from nuclei;  Activated Nuclear Factor κB (NF-κB) pathway and phosphorylation NF-κB pathway associated proteins, Iκκα, Iκκβ, NF-κBp50 and NF-κBp65 | MDCK  Influenza A virus subtype H1N1 | XSJ granules | (Yu et al., 2018) |
|  | Inhibition of RNA recipe | C6 /36 Aedes albopictus cells  Dengue virus (DENV)-1 | XSJ granules | (Zhang et al., 2019a; Yao et al., 2017a) |
|  | Interrupted SCoV-2-Spike glycoprotein binding to its receptor ACE2 and block the viral entry step | SARS-coronavirus 2 (SCoV-2) virus  Human embryonic kidney cells (HEK293T)  Kidney epithelial cells (VeroE6 and Vero cells) | Aqueous extracts of natural herb PV | (Ao et al., 2021) |
|  | Inhibition occurs primarily by interference of early, post-virion binding events | Human immunodeficiency virus (HIV)-1 | Aqueous extracts of PV | (Oh et al., 2011) |
|  | Inhibited viral gene expression | Infectious hematopoietic necrosis virus (IHNV)  Epithelioma papulosum cyprini (EPC) cells | Ursolic acid from methanol extract of PV | (Li et al., 2019a) |
|  | Bound directly to Ebola virus (EBOV)-glycoprotein pseudotyped virus and blocking the early viral events | EBOV-glycoprotein pseudotyped HIV-1-based vector system  Human umbilical vein endothelial cells (HUVECs)  Human cervical epithelial cell (HeLa)  TZM-b1 cells  Human lung carcinoma cell (A549)  Human embryonic kidney cells (HEK293T)  Kidney epithelial cells extracted from African green monkey (VeroE6) | Aqueous extract of PV | (Zhang et al., 2016) |
|  | Downregulated the expressions of LC3-II and PI3KC3 induced by DHAV and rapamycin, inhibited autophagosomes formation;  Inhibited replication | Duck hepatitis A virus (DHAV) | Polysaccharide from aqueous extracts of CI | (Ming et al., 2019) |
|  | Inhibited replication | Herpes simplex type (HSV)-1  Hepatitis A (HAV)  Vesicular stomatitis virus (VSV) | Essential oils from hydro-distillated CI | (Youssef et al., 2020) |
|  | Inhibited replication | RC-37 cells  HSV-1 strain KOS  HSV-2 strain HG52  Acyclovir-resistant strain of HSV-1 | Rosmarinic acid and caffeic acid from aqueous extracts of PV | (Nolkemper et al., 2006) |
|  | - | Human embryonic pulmonary epithelial L-132 cell line  Human coronavirus (HCoV) 229E  Single-stranded RNA viral strains (HPEV-1, HPEV-3, PV1-Lsc/2ab, and Echo11) | Caffeic acid and chlorogenic acid from aqueous and hydro methanolic extracts of MA | (Thabti et al., 2020) |
|  | Inhibited the initial stage (i.e., between virion entry and the translation of the RNA genome) in both HCV genotypes 1b and 2a | Human hepatoma Huh-7.5.1 cells  Highly HCV-JFH1-permissive subclonal cell line  Huh-7.5.1-8 cells | Caffeic acid | (Tanida et al., 2015) |
|  | Inhibition of DNA recipe  Major target exists in the early stages of infection | MDCK  HEp-2 cells and Vero cells Influenza virus A/Aichi/68 (H3N2), Sabin strain of poliovirus type 1 (PV-1) and HSV-1 strain F | Caffeic acid | (Utsunomiya et al., 2014) |
| Antioxidant | Scavenged free radicals | - | 75% alcohol extract of XSJ | (Ma et al., 2011) |
|  | Induced heme oxygenase-1(HO-1) expression | HepG2 cells | Two ursane-type triterpenes from ethanol extract of PV | (Jeong et al., 2008) |
|  | Exhibited free radical scavenging activity by the ABTS, DPPH and FRAP methods;  Increased superoxide dismutase (SOD) activity and decreased malondialdehyde (MDA) content | C57BL/6 mice | Caffeic acid, rosmarinic acid, rutin, quercetin and luteolin;  Total phenols of 60% ethanol PV extract | (Feng et al., 2010a) |
|  | Scavenged DPPH and ABTS free radicals | - | Ursolic acid and oleanolic acid from ethanol extracts of PV  Rosmarinic acid, salviaflaside, caffeic acid and hyperoside | (Chen et al., 2019) |
|  | Inhibited nitric oxide (NO) | RAW264.7 macrophages | Ursolic acid from PV | (Ryu et al., 2000) |
|  | Reduced breakage together with the apoptotic process;  Eliminated ROS production and diminished IL-6 release | Human keratinocytes HaCaT cells | Rosmarinic acid, caffeic acid from 30% ethanol extract PV | (Vostalova et al., 2010) |
|  | Induced the expression of efflux transporters through activation of Nrf2-mediated signaling pathway | HepG2 cells | Rosmarinic acid and water extract of PV | (Wu et al., 2016) |
|  | Attenuated lipopolysaccharide (LPS)-induced intracellular ROS production;  Inhibited the nuclear translocation of the nuclear factor-kappa B (NF-κB) p65 subunit and the activation of mitogen-activated protein kinases;  Diminished the phosphorylation of ERK, JNK, and p38 MAPK;  Reduced LPS-induced NO and PEG2 production and ROS accumulation | RAW 264.7 macrophages  Zebrafish | Aqueous extract of MA | (Kwon et al., 2017) |
|  | EtOAc and water fractions showed the greatest peroxyl radical-scavenging capacity and the ability to reduce Cu(I) ions;  Compounds 5-11, 18, and 19 displayed strong effects in both peroxyl radical-scavenging and reducing capacity assays; Compounds 10, 13, and 19 exhibited the most potent tartrate-resistant acid phosphatase activity in receptor activator of NF-κB ligand-induced osteoclastic | RAW 264.7 cells | Total phenolic and flavonoid from methanol extract of CI  Quercetin, luteolin, acacetin, luteolin 7-O-glucoside | (Luyen et al., 2015) |
|  | ROS scavenger;  Lipid peroxidation inhibitor;  Activated AMPK pathway | Pig sperm | Rosmarinic acid | (Elufioye and Habtemariam, 2019; Feng et al., 2020) |
|  | DPPH radical scavenging activity | - | Chlorogenic acid , rutin, and astragalin from ethanol extract of MA  Quercetin and kaempferol | (Katsube et al., 2009) |
|  | Scavenged free radicals | - | Linarin, luteolin, chlorogenic acid and apigenin from methanol extract of CI | (Hwang et al., 2016) |
|  | Modulated Nrf2-mediated oxidative stress and NF-κB-mediated inflammatory responses | C57BL/6 mice were intraperitoneal injection of streptozotocin (STZ) to induce DCM | Luteolin | (Li et al., 2019b) |
|  | Reduced nitric oxide synthase (iNOS) and cyclooxygenase-2 (COX-2) expression;  Inactivated the NF-κB signaling pathway | Sepsis-induced ALI model C57BL/6 mice | Acacetin | (Sun et al., 2018) |
|  | Inhibited lipopolysaccharide-induced inflammatory responses through modulation of NF-κB/AP-1/PI3K-Akt signaling cascades | RAW 264.7 cells | Luteolin and luteolin-7-O-glucoside | (Park and Song, 2013) |
|  | DPPH radical scavenging activity;  ABTS radical scavenging activity;  FRAP antioxidant activity | RAW 264.7 cells | Quercetin and luteolin from ethanol extract of CI | (Kang et al., 2021) |
|  | Inhibited the STAT3 pathway to antiproliferative  Modulated of Nrf2/MAPK mediated HO-1 signaling | RAW 264.7 cells  HUVEC cells | Luteolin 7-O-glucoside | (De Stefano et al., 2021; Song and Park, 2014) |
|  | Activation of Nrf-2 through PI3K/Akt signaling pathway | Myocardial cell H9C2  Langendorff heart model | Linarin | (Yu et al., 2017) |
| Anticancer | Inhibited the growth of proximal tubular epithelial cells (PTC) *in vivo* and *in vitro* via autophagy, which is associated with the AMPK/mTOR/ULK1 pathway | BALB/c mice xenograft model and the human PTC cell line K1  Normal human thyroid follicular epithelial cells | Aqueous extract of PV | (Song et al., 2021a) |
|  | Inhibited the proliferation and migration of thyroid cancer (TC) cells both *in vitro* and *in vivo*, which may have been achieved via inhibiting the expression of MKI67 and PCNA and promoting the expression of CDH1 | Human papillary TC cell lines  Human squamous TC cell line  Human follicular TC cell line  Female Balb/c nude mice xenotransplanted tumor model | 50% ethanol extract of PV | (Yu et al., 2021) |
|  | Inhibition of autophagy and promotion of apoptosis, which is related to activation of the PI3K/Akt/mTOR pathway | SMMC-7721 cells  H22 tumor bearing mice | Total flavonoids from 75% ethanol extract of PV | (Song et al., 2021b) |
|  | Suppression of angiogenesis, induction of apoptosis, cell cycle arrest and modulation of PI3K/AKT signaling pathway | Human breast cancer cell line MCF-5  Xenografted mice models | Methanolic root extract of PV | (Gao and Xu, 2019) |
|  | - | SW579 cells | Pentacyclic triterpenoids from 95% ethanol extract of PV | (Zheng et al., 2022) |
|  | p53-mediated apoptosis | Induced pluripotent stem cells (iPSCs) | Ethanol extract of PV | (Kim et al., 2020) |
|  | Activated the proapoptotic protein caspase-3 and induced cellular apoptotic pathway | Lung cancer cells A549 | Ethanol extract of PV | (Zhu et al., 2018) |
|  | Induced apoptosis through suppression of constitutive STAT3 activation in parallel with the inhibition of constitutive JAK1 and JAK2 activation;  Down modulated the expression of STAT3-regulated gene products, including Bcl-xl, Bcl-2, IAP-1, cyclin D1, and survivin, and caused cytotoxicity, accumulation of cells in the G1/G0 phase, and apoptosis through caspase-3 activation | Human prostate cancer DU145 cells  Human breast carcinoma MDA-MB-231  Human multiple myeloma cell lines U266 | Acacetin from ethanol extract of CI | (Kim et al., 2013) |
|  | Induced factor-related apoptosis-induced ligand (TRAIL)-regulated apoptosis both through the intrinsic and extrinsic pathways via the up-regulation of Caspases | Human glioma cell lines of A-172, U343, U87MG, and T98G cells  Mouse microglia of BV2  Balb/c athymic (nu/nu) mice | Linarin from CI | (Xu et al., 2017) |
|  | Inhibition of NF-κB/p65 and up-regulating p53 expression | Human glioma cell lines, U87-MG and U251  Human normal glia cell line of HEB  Human normal liver cell line of HL-7702，H4, A172  Mouse microglia of BV2 | Linarin from CI | (Zhen et al., 2017) |
|  | Increased the TNF-α content | Human lung adenocarcinoma A549 and SPC-A-1 cells  Mice lung adenocarcinoma Lewis cells  Male C57BL/6 mice | Caffeic acid, rosmarinic acid, rutin, quercetin, oleanolic acid and ursolic acid.  Total triterpenes and total phenols from 95% ethanol extract of PV | (Feng et al., 2010b) |
|  | - | Lymphocytic leukemia cells P-388 and L-1210  Human lung carcinoma cell A-549  Human colon (HCT-8) and mammary (MCF-7) tumor cells | Ursolic Acid from PV | (Lee et al., 1988) |
|  | Recovered the effects of endoplasmic reticulum stress-induced resistance to DOX through COX-2 or p38 MAPK-mediated inactivation of the PI3K/Akt pathway | HepG2 | Chlorogenic acid, rutin, quercetin, astragalin, and kaempferol from methanol polyphenol extract of MA | (Yang et al., 2020) |
|  | Apoptosis by the activation of extrinsic caspase-dependent pathway by upregulating the mRNA expressions of caspase-3, caspase-8, and TNF-α. Induces intrinsic apoptosis pathway as evidenced by the induction of cytochrome c, Bax, and caspase-3 | Human prostate cancer PC-3 cells | Apigenin | (Imran et al., 2020) |
|  | Methanol and water extracts dose dependently inhibited mushroom tyrosinase activity, and the effects of methanol extract were similar to those of kojic acid, a well-known tyrosinase inhibitor | Human skin | Luteolin and acacetin-7-O-rutinoside from water extract of CI | (Choi et al., 2016) |
|  | Inhibited cell growth, dose- and time-dependently, but did not cause apoptosis;  Accumulation of treated cells in G2/M phase;  Induced granulocytic differentiation | HL-60 cells  Breast cancer cell lines (MCF-7 and MDA-MB-231) | Apigenin 7-O-glucoside | (Nakazaki et al., 2013; Goodarzi et al., 2020) |
| Anti-diabetic | Attenuated IL-1beta-increased NF-κB binding activity and inflammatory cytokine expression | INS-1 cells | Aqueous extract of PV | (Wu et al., 2012) |
|  | Disruption of the TGF-β/Smad signaling | Human mesangial cell (HMC)  diabetic rats | Aqueous extract of PV | (Namgung et al., 2017) |
|  | Increased serum-insulin, attenuation of α-amylase and α-glucosidase | Alloxan-induced diabetic mice  Alloxan-induced type 1 diabetes (T1D) mouse model | Rosmarinic acid, caffeic acid, rutin and quercetin form 80% ethanol of PV | (Raafat et al., 2016) |
|  | Improved glucose metabolism disorders, decreased insulin resistance, and ameliorated the antioxidative ability | Type 2 diabetes mellitus (T2DM) rat model | Aqueous extract of MA | (Lyu et al., 2021) |
|  | Up-regulaed the expression of p-AMPKα, down-regulated the expression of PPARγ,C/EBPα and LPL;  Inhibited the differentiation of preadipocytes into mature fat cells,and reduced the volume of fat cells | Diet-induced obesity rat | MA extract | (Wu et al., 2017) |
|  | Insulin resistance via IRS-1/PI3K/Glut-4 signalling pathway | T2DM rats | Aqueous extract of MA | (Cai et al., 2016) |
|  | Improved the glucose tolerance and lower the level of glucose | Male ICR mice and BALB/c mice  Streptozocin-induced diabetic mice | Chlorogenic acid, caffeic acid, rutin, isoquercitrin astragalin, and dicaffeoylquinic acid from 75% ethanol extract of MA | (Ma et al., 2016) |
|  | Activated AMPK pathway and down-regulated transcription factors required for adipogenesis, such as C/EBPs and PPARs | 3T3-L1 preadipocyte  High-fat-diet-induced obese C57BL/6 mice mice | 50% ethanol or distilled water extract of CI | (Nepali et al., 2018) |
|  | Improved the fat metabolism and cell size in S6k and Akt1 mutant flies | *Drosophila melanogaster* were fed with high-sugar diet | Aqueous extract of CI | (Bai et al., 2018) |
|  | Inhibited the formation of advanced glycation end products (AGEs) and Nε-(carboxymethyl)lysine (CML) | Glycation model | Acacetin, apigenin, chlorogenic acid, kaempferol, luteolin and quercetin from water extract of CI | (Tsuji-Naito et al., 2009) |
|  | Controlled blood glucose and antioxidants;  Decreased in SOD mRNA expression in pancreatic β cells | Streptozotozin (STZ)-diabetic rats | Triterpenes acid and ursolic acid from 75% ethanol extract of PV | (Zhou et al., 2013) |
|  | Exhibited a hypocholesterolemic and hypotriacylglyceridemic effect attributed to the stimulatory effect on the β-oxidation of fatty acids;  Induction of fatty acid oxidation, inhibition of lipogenesis, and suppression of oxidative stress | Rats fed a high-fat diet | Total polyphenols, total flavonoid aglycons, quercetin and kaempferol from water extract of MA | (Kobayashi et al., 2010) |
|  | Lowered plasma glucose levels with tendencies for dose dependency | T2D Rats | Chlorogenic acid and rutin from 70% ethanol extract of MA | (Hunyadi et al., 2012) |
|  | Improved lipid and glucose metabolism, attenuated oxidative stress and enhanced β-oxidation | Male C57BL/6N mice were fed a high-fat diet | Rutin and quercetin derivatives from MA | (Sun et al., 2015) |
|  | Reduced DAG-PKC cascades including regulating energy metabolism, improving insulin sensibility and reducing inflammatory response | Sprague-Dawley male rats with high-fat diet or AIN93G diet  STZ induced diabetic nephropathy rats | Rutin, chlorogenic acid, β-sitosterol, flavonoids and polyphenols from MA powder | (Sheng et al., 2018) |
|  | Activation of IR and GLUT4;  Attenuated insulin resistance in adipose tissue via IRS-1/Akt mediated insulin signaling | High fat and sucrose-induced type-2 diabetic rats | β-Sitosterol | (Ponnulakshmi et al., 2019; Babu et al., 2020) |
| Antibacterial | - | *Staphylococcus aureus*  *Streptococcus haemolyticus* | XSJ granules | (Guo, 2010) |
|  | - | *Escherichia coli (E. coli)*  Urine samples of forty four patients | Aqueous extract and ethanol extract of PV | (Komal et al., 2018) |
|  | - | *E. coli*  *Klebsiella pneumoniae*  *Providencia stuartii*  *Pseudomonas aeruginosai*  *methicillin-resistant Staphylococcus aureus species* | Leaves and flower extracts (aqueous and 70% methanolic) of PV | (Grosan et al., 2020) |
|  | The 50% v/v and 80% v/v methanol extracts and fractions F3, F4 and FA are richer in phenolic compounds that showed antibacterial activity;  Methanolic extracts have better antimicrobial activity than other eluents | Gram-positive bacteria  *Streptococcus aureus*  *Streptococcus pneumoniae*  *Streptococcus epidermidis*  *Bacillus cereus*  *Bacillus subtilis* | Chlorogenic acid, caffeic acid, protocatechuic acid, luteolin-7-glucoside, apigenin-7-glucoside, kaempferol-3-glucoside, linarin, apigenin, luteolin and kaempferol from methanol extract of CI | (Kozyra et al., 2015) |
|  | - | *Klebsiella pneumonia*  *E. coli,*  *Pseudomonas aeruginosa*  3T3 mouse embryo fibroblast cells | Silver nanoparticles (AgNPs) of CI aqueous extract  Flavonoids, terpenoids, and glycosides | (Arokiyaraj et al., 2014) |
|  | - | Gram-positive bacteria  *Staphylococcus aureus*  *Enterococcus faecalis*  *Streptococcus agalactiae*  *Streptococcus oralis*  *Streptococcus intermedius*  *Streptococcus gordonii*  HaCaT cells | Rosmarinic acid, caffeic acid from total methanol extract of PV | (Psotova et al., 2003) |
|  | - | *Porphyromonas gingivalis*  *Prevotella intermedia*  *Fusobacterium nucleatum*  *Aggregatibacter actinomycetemcomitans*  KB cells | Oleanolic acid and ursolic acid methanol extract of MA | (Park et al., 2014) |
|  | Compromise the integrity of the bacterial membrane;  Protein synthesis inhibitor that interferes with translation accuracy–induced Clp proteins known to be induced by misfolded proteins;  Elicit the oxidative response in MRSA via the AhpC induction | Methicillin-Resistant Staphylococcus aureus (MRSA) | Ursolic acid | (Wang et al., 2016) |
|  | Inhibited bacterial enzyme activity, including respiratory enzymes;  Produce ROS when subjected to light | E. coli O157:H7, Salmonella Typhimurium, and L. monocytogenes. | Caffeic acid | (Park and Kang, 2021) |
|  | Damage of intracelluar and outer membranes as well as disruption of cell metabolism resulted in cell death | Salmonella Enteritidis S1 | Chlorogenic acid | (Sun et al., 2020) |
|  | - | Staphylococcus aureus, Escherichia coli | β- Sitosterol | (Ododo et al., 2016) |
| Immunomodulatory effect | Inhibited Th1/Th17 immune responses | Experimental autoimmune thyroiditis (EAT) rat model | Aqueous extract of PV | (Zhu et al., 2022) |
|  | Inhibited HMGB1/TLR9 signaling involved in the PV-mediated reductions in Th1, Th2 and Th17 cells | Rat model of thyroglobulin-induced EAT and in LPS-induced thyroid follicular cells (TFCs) | Rosmarinic acid from aqueous extract of PV | (Guo et al., 2021) |
|  | Suppressed Con A-, LPS- and OVA-induced splenocyte proliferation in the immunized mice;  Total IgG, IgG1 and IgG2b levels were significantly reduced;  Suppressed the cellular and humoral response | ICR mice | Triterpenoids, flavonoids, tannins and polysaccharide from ethanol extract of PV | (Sun et al., 2005) |
|  | Immuno-stimulated activities involving TLR2, TLR4 and CR3 | RAW 264.7 cells | Heteropolysaccharide from water extract of PV | (Li et al., 2015) |
|  | Suppressed the activation of NF-κB and interferon regulatory factor 3 (IRF3), and their downstream corresponding promoters;  Inhibited genes related to antigen presentation pathways induced by dsDNA or dsRNA | Pathogen-associated molecular patterns (PAMP)/ danger-associated molecular patterns (DAMP)-stimulated rat thyroid FRTL-5 cells | Ursolic acid, quercetin, β-sitosterol from aqueous extract of PV | (Chen et al., 2020a) |
|  | Stimulated the production of NO and PGE2 as immune response parameters at noncytotoxic concentrations;  Associated with the increased expression of inducible NO synthase and COX-2. | Murine RAW264.7 macrophages | Water extract of MA | (Kwon et al., 2016) |
|  | Increased the delayed-type hypersensitivity (DTH) reaction induced by 2,4-dinitro-fluorobenzene (DNFB);  Enhanced antibody generation and IgG and IgM levels in mice sera in response to sheep red blood cells (SRBC) | Splenic cells of mice  cyclophosphamide (CP)-induced mice. | Butanol soluble fraction from ethanol extract of CI | (Cheng et al., 2005) |
|  | Increased viable peripheral blood mononuclear cell (PBMC) numbers and activated swine dendritic cells (DCs);  Drove the BMDC response towards a Th1 pattern with IFN-α secretion and absence of IL-10 | Peripheral blood mononuclear cells (PBMCs) & Bone marrow hematopoietic cells (BMHCs)  Male Pig | β-sitosterol | (Fraile et al., 2012) |
| Hepatoprotective activity | Hepatoprotective potential and free radical scavenging | Male Albino Wistar rats | Flavonoids and total phenolic from methanolic, ethanolic, hydroalcoholic and aqueous extracts of PV | (Ahmad et al., 2020) |
|  | Reduced the contents of inflammatory factors(TNF-alpha, IL-6 and IL-1beta) and liver function markers(ALT, AST, ALP);  Improved metabolic disorder of liver injury induced by ethanol, and amino acids, fatty acids, and phospholipids | Female Wistar rats | 80% methanol extract of PV | (Deng et al., 2021) |
|  | Increased the suppressed body weight, attenuated the decline of thymus and spleen indexes, and reduced the elevated levels of ALT and AST;  Alleviated D-gal-induced abnormal alterations in structure and function of brain and liver dose-dependently via renewing normal antioxidant enzymes activities (SOD, CAT, GSH-Px), reducing MDA accumulation, decreasing inflammatory cytokines productions (IL-1beta, IL-6, TNF-alpha), as well as attenuating the increase of Bax/Bcl-2 ratio and cleaved caspase-3 activation in the liver and brain | Mice injected with D-galactose. | Supercritical carbon dioxide fluid extract from flowers and buds of CI | (Zhang et al., 2019b) |
|  | Inhibited bioactivation of CCl4-induced hepatotoxicity and downregulated CYP2E1 expression | Normal human hepatocytes (Chang cell)  Hepatocellular carcinoma cells (HepG2) | Water extract from CI | (Jeong et al., 2013) |
|  | Scavenged or reduced activities-superoxide or peroxynitirite rather than to inhibit TNF-production;  Reduced the elevation of plasma AST levels, as well as anti-TNF and SOD. Reduced the expression of TNF-αmRNA in liver and plasma | D-galactosamine (D-GalN)-sensi- tized mice | Rosmarinic acid | (Osakabe et al., 2002) |
|  | Reduction in the serum concentration of AST, ALT, and LDH | Liver I/R rats model | Rosmarinic acid | (Rocha et al., 2015) |
|  | Reduced in granuloma size, production of nitric oxide and malondialdehyde, and glutathione levels;  Prevented the increase in the hepatic malondialdehyde;  Antifibrotic role | *Schistosoma mansoni* cercariae infected Swiss albino mice | β-Sitosterol, rutin and isoquercitrin from 70% methanol MA extract | (Amer et al., 2013) |
|  | Downregulated the expression of apoptosis-related genes through the PI3K/Akt pathway. | Male Wistar rats | β-sitosterol | (Chen et al., 2020b) |
|  | Restored the liver enzymes (ALT and AST), liver lipid peroxidation markers (MDA and CAT), total bilirubin and albumin to their normal levels without inhibitory effect on the CYP2E1 activity | Male Sprague-dawley rats | β-sitosterol | (Abdou et al., 2019) |
|  | Suppressed the elevation of GPT, GOT, MDA and 8-OHdG, and inhibited the reduction of GSH in a dose-dependent manner *in vivo*, but also reduced the damage of hepatocytes *in vitro*. antioxidant properties, acting as scavengers of ROS | Male Wistar rats | Apigenin-7-glucoside | (Zheng et al., 2005) |
|  | Accelerated liver proliferation, recovered liver function, and protected the integrity of hepatocytes against liver damage;  Activation of C/EBPβ expression | C57BL/6 male mice | Oleanolic acid and ursolic acid from PV | (Liu, 1995; Jin et al., 2012) |
|  | Reprogrammed the liver to activate the Nrf2 | BALB/c mice | Oleanolic acid | (Liu et al., 2019) |


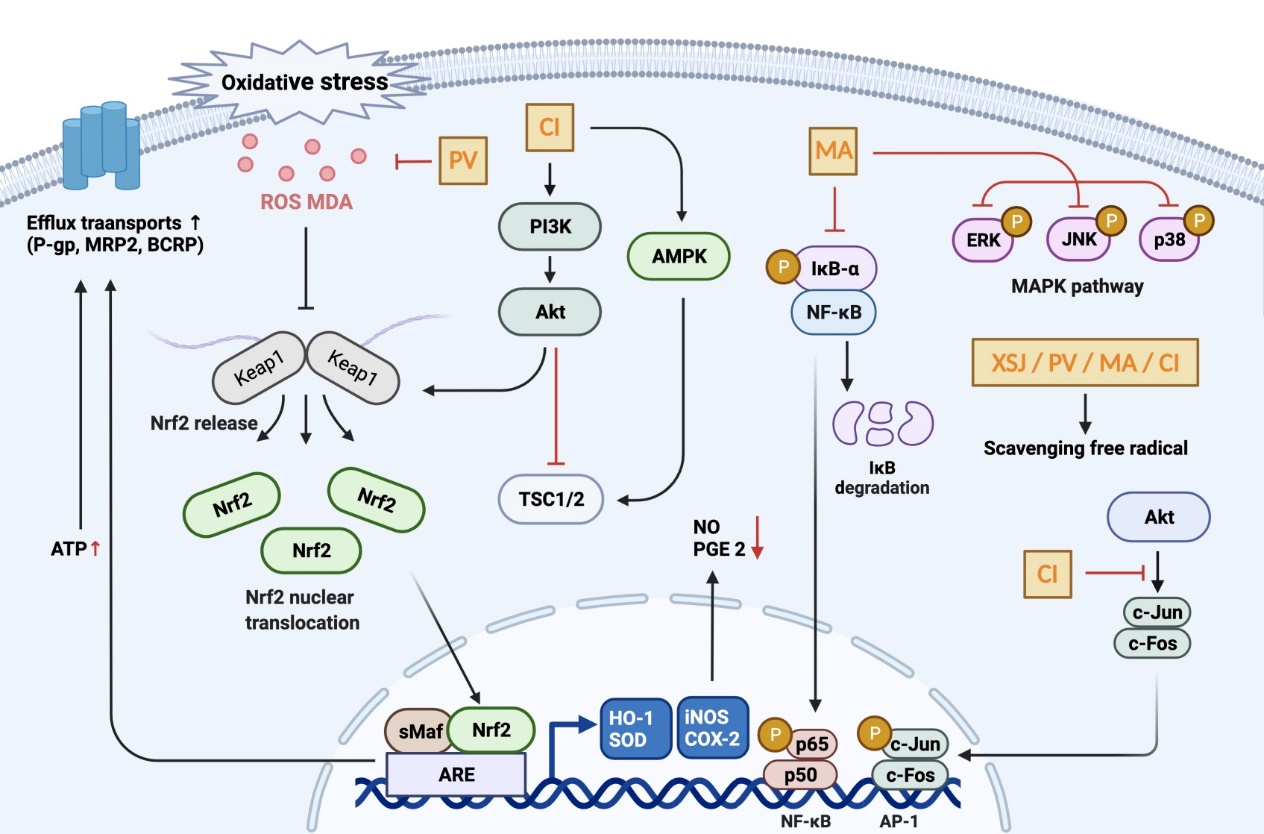


**Figure 4.** Schematic of the molecular mechanisms of XSJ in antioxidant effect.


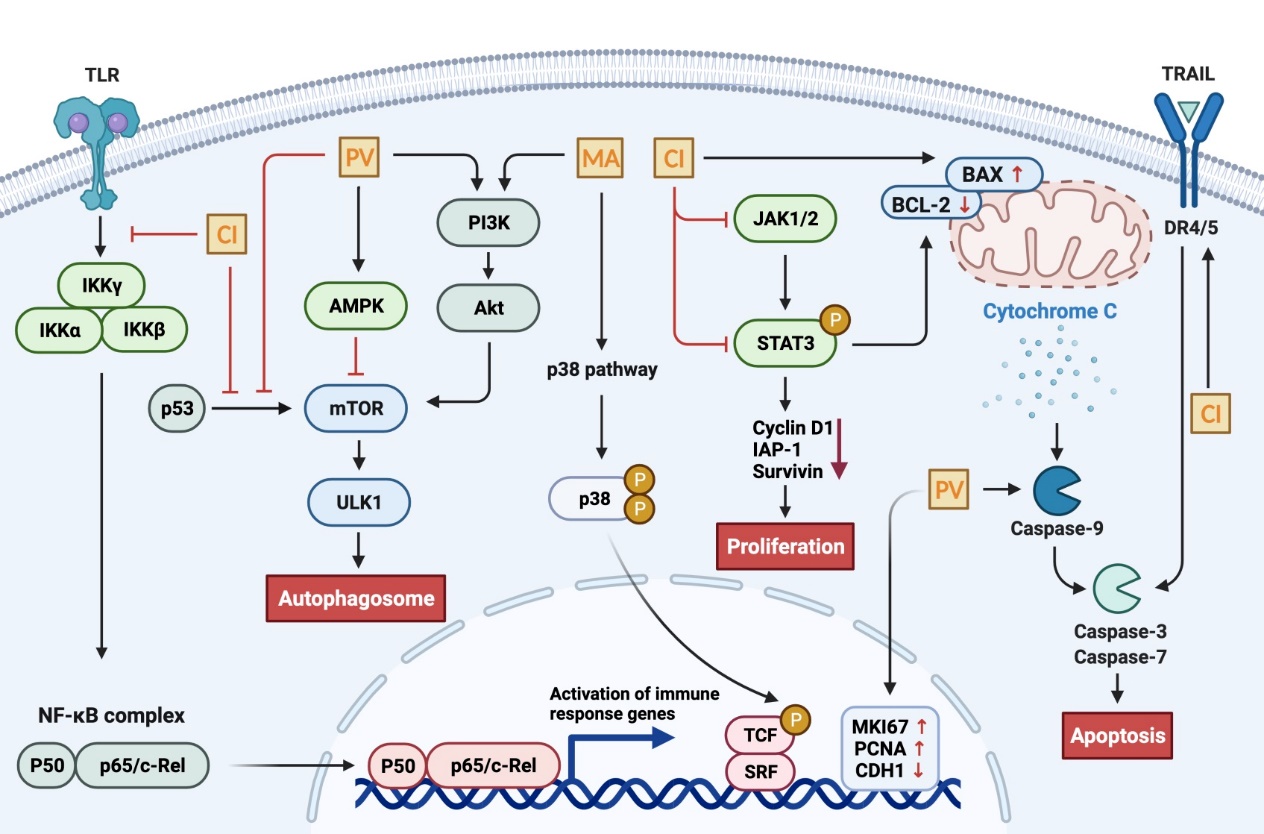


**Figure 5.** Schematic of the molecular mechanisms of XSJ in autophagosome, apoptosis and antiproliferative activity.


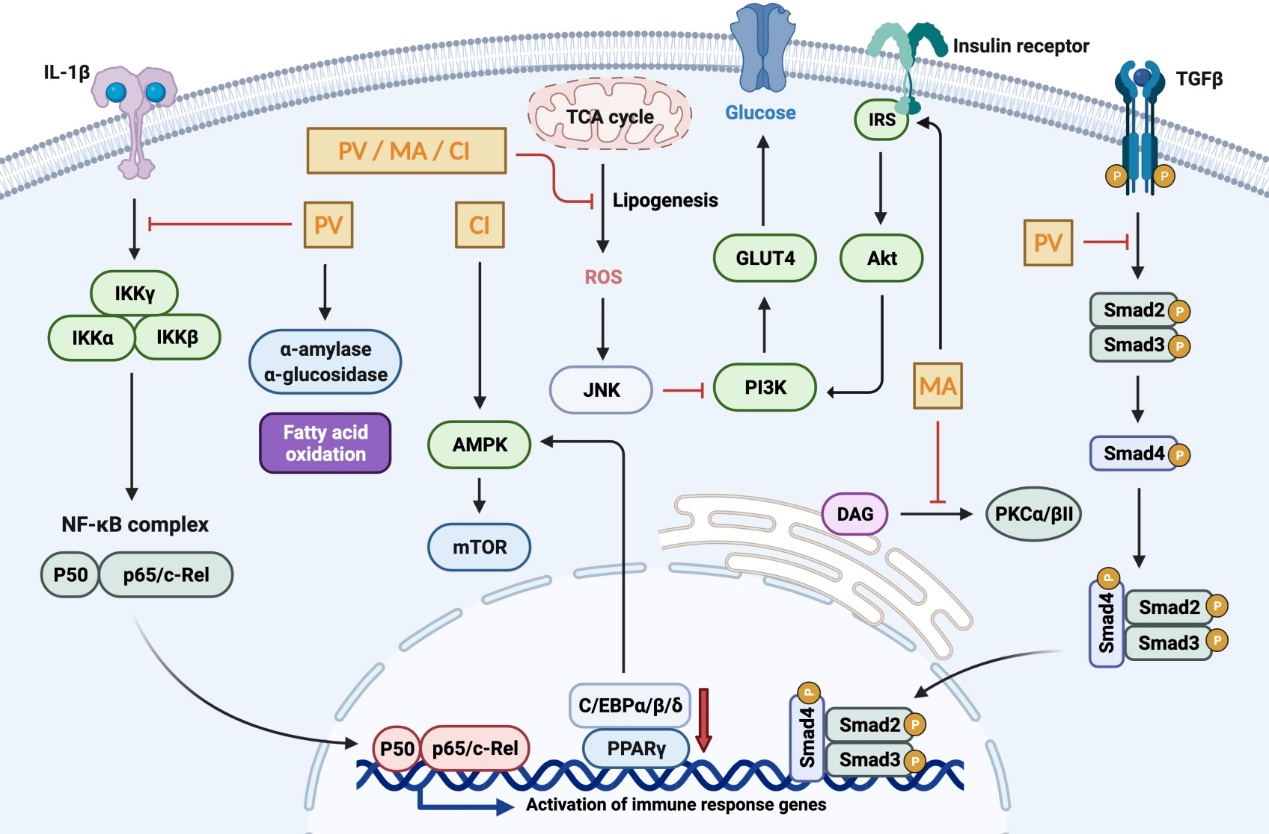


**Figure 6.** Schematic of the molecular mechanisms of XSJ in antidiabetic activity.


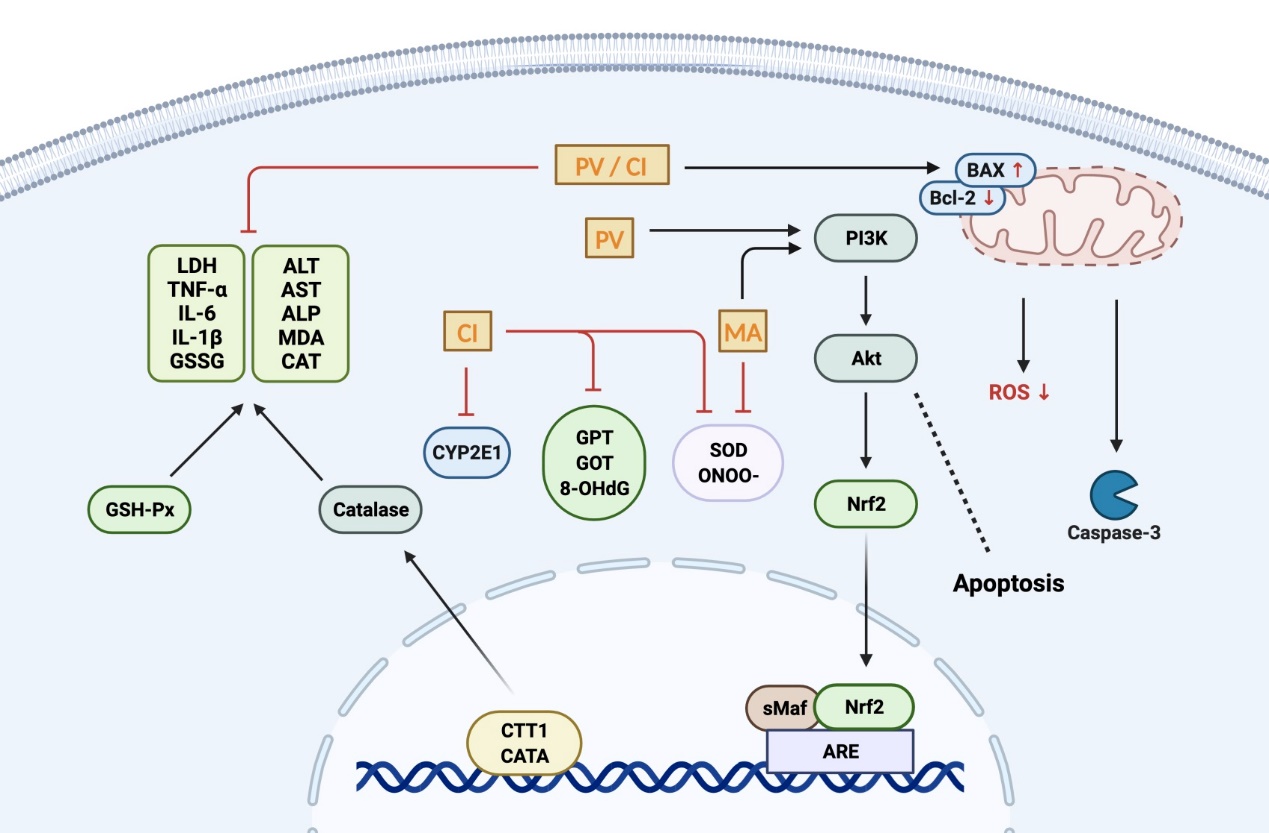


**Figure 7.** Schematic of the molecular mechanisms of XSJ in hepatoprotective activity.

**Reference:**

Abdou E. M., Fayed M. A. A., Helal D., Ahmed K. A. (2019). Assessment of the hepatoprotective effect of developed lipid-polymer hybrid nanoparticles (LPHNPs) encapsulating naturally extracted beta-Sitosterol against CCl4 induced hepatotoxicity in rats. *Sci Rep.* 9, 19779. 10.1038/s41598-019-56320-2

Ahmad G., Masoodi M. H., Tabassum N., Mir S. A., Iqbal M. J. (2020). Invivo hepatoprotective potential of extracts obtained from floral spikes of Prunella vulgaris L. *J Ayurveda Integr Med.* 11, 502-507. 10.1016/j.jaim.2019.08.003

Amer O. S. O., Dkhil M. A., A-Quraishy S. (2013). Antischistosomal and Hepatoprotective Activity of Morus alba Leaves Extract. *Pakistan Journal of Zoology.* 45, 387-393.

Ao Z., Chan M., Ouyang M. J., Olukitibi T. A., Mahmoudi M., Kobasa D., et al. (2021). Identification and evaluation of the inhibitory effect of Prunella vulgaris extract on SARS-coronavirus 2 virus entry. *PLoS One.* 16, e0251649. 10.1371/journal.pone.0251649

Arokiyaraj S., Arasu M. V., Vincent S., Prakash N. U., Choi S. H., Oh Y. K., et al. (2014). Rapid green synthesis of silver nanoparticles from Chrysanthemum indicum L and its antibacterial and cytotoxic effects: an in vitro study. *Int J Nanomedicine.* 9, 379-88. 10.2147/IJN.S53546

Babu S., Krishnan M., Rajagopal P., Periyasamy V., Veeraraghavan V., Govindan R., et al. (2020). Beta-sitosterol attenuates insulin resistance in adipose tissue via IRS-1/Akt mediated insulin signaling in high fat diet and sucrose induced type-2 diabetic rats. *Eur J Pharmacol.* 873, 173004. 10.1016/j.ejphar.2020.173004

Bai Y., Li K., Shao J., Luo Q., Jin L. H. (2018). Flos Chrysanthemi Indici extract improves a high-sucrose diet-induced metabolic disorder in Drosophila. *Exp Ther Med.* 16, 2564-2572. 10.3892/etm.2018.6470

Cai S.-f., Shi J., Huang X., Huang H. (2014). Xiasangju fufang peiwu de huaxuechengfen bianhua yanjiu [Study on the chemical composition variation of Xiasangju compound compounding]. *Journal of North Pharmacy.* 11, 10-11.

Cai S. Y., Sun W., Fan Y. X., Guo X., Xu G. Y., Xu T. H., et al. (2016). Effect of mulberry leaf (Folium Mori) on insulin resistance via IRS-1/PI3K/Glut-4 signalling pathway in type 2 diabetes mellitus rats. *Pharmaceutical Biology.* 54, 2685-2691. 10.1080/13880209.2016.1178779

Chen F., Kawashima A., Luo Y., Kiriya M., Suzuki K. (2020a). Innate Immune-Modulatory Activity of Prunella vulgaris in Thyrocytes Functions as a Potential Mechanism for Treating Hashimoto's Thyroiditis. *Front Endocrinol (Lausanne).* 11, 579648. 10.3389/fendo.2020.579648

Chen Y., Zhang X., Guo Q., Cao L., Qin Q., Li C., et al. (2019). Plant morphology, physiological characteristics, accumulation of secondary metabolites and antioxidant activities of Prunella vulgaris L. under UV solar exclusion. *Biol Res.* 52, 17. 10.1186/s40659-019-0225-8

Chen Z. J., Wu A. C., Jin H. M., Liu F. H. (2020b). beta-Sitosterol attenuates liver injury in a rat model of chronic alcohol intake. *Archives of Pharmacal Research.* 43, 1197-1206.

Cheng W., Li J., You T., Hu C. (2005). Anti-inflammatory and immunomodulatory activities of the extracts from the inflorescence of Chrysanthemum indicum Linne. *J Ethnopharmacol.* 101, 334-7. 10.1016/j.jep.2005.04.035

Choi K. T., Kim J. H., Cho H. T., Lim S. S., Kwak S. S., Kim Y. J. (2016). Dermatologic evaluation of cosmetic formulations containing Chrysanthemum indicum extract. *J Cosmet Dermatol.* 15, 162-8. 10.1111/jocd.12211

De Stefano A., Caporali S., Di Daniele N., Rovella V., Cardillo C., Schinzari F., et al. (2021). Anti-Inflammatory and Proliferative Properties of Luteolin-7-O-Glucoside. *International Journal of Molecular Sciences.* 22.

Deng J., Li L., Lin L. M., Li Y. M., Xia B. H., Liao D. F. (2021). [Metabolic mechanism of Prunella vulgaris in treatment of ethanol-induced oxidative stress in rats based on metabonomics]. *Zhongguo Zhong Yao Za Zhi.* 46, 1813-1821. 10.19540/j.cnki.cjcmm.20210122.503

Elufioye T. O., Habtemariam S. (2019). Hepatoprotective effects of rosmarinic acid: Insight into its mechanisms of action. *Biomedicine & Pharmacotherapy.* 112.

Feng L., Jia X., Zhu M. M., Chen Y., Shi F. (2010a). Antioxidant activities of total phenols of Prunella vulgaris L. in vitro and in tumor-bearing mice. *Molecules.* 15, 9145-56. 10.3390/molecules15129145

Feng L., Jia X. B., Jiang J., Zhu M. M., Chen Y., Tan X. B., et al. (2010b). Combination of active components enhances the efficacy of Prunella in prevention and treatment of lung cancer. *Molecules.* 15, 7893-906. 10.3390/molecules15117893

Feng T. Y., Lv D. L., Zhang X., Du Y. Q., Yuan Y. T., Chen M. J., et al. (2020). Rosmarinic acid improves boar sperm quality, antioxidant capacity and energy metabolism at 17 degrees C via AMPK activation. *Reproduction in Domestic Animals.* 55, 1714-1724.

Fraile L., Crisci E., Cordoba L., Navarro M. A., Osada J., Montoya M. (2012). Immunomodulatory properties of beta-sitosterol in pig immune responses. *Int Immunopharmacol.* 13, 316-21. 10.1016/j.intimp.2012.04.017

Gao W., Xu H. (2019). Root extract of Prunella vulgaris inhibits in vitro and in vivo carcinogenesis in MCF-5 human breast carcinoma via suppression of angiogenesis, induction of apoptosis, cell cycle arrest and modulation of PI3K/AKT signalling pathway. *J BUON.* 24, 549-554.

Goodarzi S., Tabatabaei M. J., Mohammad Jafari R., Shemirani F., Tavakoli S., Mofasseri M., et al. (2020). Cuminum cyminum fruits as source of luteolin- 7-O-glucoside, potent cytotoxic flavonoid against breast cancer cell lines. *Nat Prod Res.* 34, 1602-1606. 10.1080/14786419.2018.1519824

Grosan A., Vari C. E., Stefanescu R., Danciu C., Pavel I. Z., Dehelean C., et al. (2020). Antibacterial and antitumor activity of the species Prunella vulgaris L. *Revista Romana De Medicina De Laborator.* 28, 405-417. 10.2478/rrlm-2020-0031

Guo J. (2010). Yizhong Xiasangju guodong [A kind of summer Sang Ju Yin jelly]. CHINA Patent CN101874574A.

Guo Q., Qu H., Zhang H., Zhong X. (2021). Prunella vulgaris L. Attenuates Experimental Autoimmune Thyroiditis by Inhibiting HMGB1/TLR9 Signaling. *Drug Des Devel Ther.* 15, 4559-4574. 10.2147/DDDT.S325814

Hua R.-f., Yao J.-x., Li Z.-j., Fang T.-z., Jiang L.-j., Xu Z.-d. (2013). HPLC tongshi ceding Xiasangju keli zhong midiexiangsuan yu yimidiexiangsuangan de hanliang [Simultaneous Determination of Ｒosmarinic Acid and Salviaflaside in Xiasangju Granules of by HPLC]. *Chinese Journal of Experimental Traditional Medical Formulae.* 19, 75-77.

Huang X.-j., Hou W., Zhao Y.-l., Luo F., Yang Z.-q. (2007). Xiasangju kang huxidaohebao bingdu de shiyan yanjiu [Experimental study on the anti-respiratory syncytial virus of Xiasangju]. *Chinese Journal of Modern Drug Application* 11-14.

Hunyadi A., Martins A., Hsieh T. J., Seres A., Zupko I. (2012). Chlorogenic Acid and Rutin Play a Major Role in the In Vivo Anti-Diabetic Activity of Morus alba Leaf Extract on Type II Diabetic Rats. *Plos One.* 7.

Hwang S. H., Paek J. H., Lim S. S. (2016). Simultaneous Ultra Performance Liquid Chromatography Determination and Antioxidant Activity of Linarin, Luteolin, Chlorogenic Acid and Apigenin in Different Parts of Compositae Species. *Molecules.* 21. 10.3390/molecules21111609

Imran M., Gondal T. A., Atif M., Shahbaz M., Qaisarani T. B., Mughal M. H., et al. (2020). Apigenin as an anticancer agent. *Phytotherapy Research.* 34, 1812-1828.

Jeong G. S., An R. B., Pae H. O., Oh G. S., Chung H. T., Kim Y. C. (2008). Heme oxygenase-1 inducing constituent of Prunella vulgaris in HepG2 cells. *Biol Pharm Bull.* 31, 531-3. 10.1248/bpb.31.531

Jeong S. C., Kim S. M., Jeong Y. T., Song C. H. (2013). Hepatoprotective effect of water extract from Chrysanthemum indicum L. flower. *Chin Med.* 8, 7. 10.1186/1749-8546-8-7

Jin Y. R., Jin J. L., Li C. H., Piao X. X., Jin N. G. (2012). Ursolic acid enhances mouse liver regeneration after partial hepatectomy. *Pharm Biol.* 50, 523-8. 10.3109/13880209.2011.611143

Kang H., Park C.-H., Kwon S.-O., Lee S.-G. (2021). Antioxidant and anti-inflammatory activities of Chrysanthemum indicum Linne extracts at different ethanol ratios. *Korean Journal of Food Science and Technology.* 53, 416-422.

Katsube T., Tsurunaga Y., Sugiyama M., Furuno T., Yamasaki Y. (2009). Effect of air-drying temperature on antioxidant capacity and stability of polyphenolic compounds in mulberry (Morus alba L.) leaves. *Food Chemistry.* 113, 964-969.

Kim A., Lee S. Y., Seo C. S., Chung S. K. (2020). Prunellae Spica Extract Suppresses Teratoma Formation of Pluripotent Stem Cells through p53-Mediated Apoptosis. *Nutrients.* 12. 10.3390/nu12030721

Kim C., Kim M. C., Kim S. M., Nam D., Choi S. H., Kim S. H., et al. (2013). Chrysanthemum indicum L. extract induces apoptosis through suppression of constitutive STAT3 activation in human prostate cancer DU145 cells. *Phytother Res.* 27, 30-8. 10.1002/ptr.4689

Kobayashi Y., Miyazawa M., Kamei A., Abe K., Kojima T. (2010). Ameliorative effects of mulberry (Morus alba L.) leaves on hyperlipidemia in rats fed a high-fat diet: induction of fatty acid oxidation, inhibition of lipogenesis, and suppression of oxidative stress. *Biosci Biotechnol Biochem.* 74, 2385-95. 10.1271/bbb.100392

Komal S., Kazmi S. A. J., Khan J. A., Gilani M. M. (2018). Antimicrobial activity of Prunella Vulgaris extracts against multi-drug resistant Escherichia Coli from patients of urinary tract infection. *Pak J Med Sci.* 34, 616-620. 10.12669/pjms.343.14982

Kozyra M., Biernasiuk A., Malm A., Chowaniec M. (2015). Chemical compositions and antibacterial activity of extracts obtained from the inflorescences of Cirsium canum (L.) all. *Natural Product Research.* 29, 2059-2063.

Kwon D. H., Cheon J. M., Choi E. O., Jeong J. W., Lee K. W., Kim K. Y., et al. (2016). The Immunomodulatory Activity of Mori folium, the Leaf of Morus alba L., in RAW 264.7 Macrophages In Vitro. *J Cancer Prev.* 21, 144-151. 10.15430/JCP.2016.21.3.144

Kwon D. H., Jeong J. W., Choi E. O., Lee H. W., Lee K. W., Kim K. Y., et al. (2017). Inhibitory effects on the production of inflammatory mediators and reactive oxygen species by Mori folium in lipopolysaccharide-stimulated macrophages and zebrafish. *Anais Da Academia Brasileira De Ciencias.* 89, 661-674. 10.1590/0001-3765201720160836

Lee K. H., Lin Y. M., Wu T. S., Zhang D. C., Yamagishi T., Hayashi T., et al. (1988). The cytotoxic principles of Prunella vulgaris, Psychotria serpens, and Hyptis capitata: ursolic acid and related derivatives. *Planta Med.* 54, 308-11. 10.1055/s-2006-962441

Li B. Y., Hu Y., Li J., Shi K., Shen Y. F., Zhu B., et al. (2019a). Ursolic acid from Prunella vulgaris L. efficiently inhibits IHNV infection in vitro and in vivo. *Virus Res.* 273, 197741. 10.1016/j.virusres.2019.197741

Li C., You L., Fu X., Huang Q., Yu S., Liu R. H. (2015). Structural characterization and immunomodulatory activity of a new heteropolysaccharide from Prunella vulgaris. *Food Funct.* 6, 1557-67. 10.1039/c4fo01039f

Li L., Luo W., Qian Y. Y., Zhu W. W., Qian J. C., Li J. L., et al. (2019b). Luteolin protects against diabetic cardiomyopathy by inhibiting NF-kappa B-mediated inflammation and activating the Nrf2-mediated antioxidant responses. *Phytomedicine.* 59.

Lin L.-m., Xia B.-h., Liu J.-y., Li C., He Y.-c., Yao J.-x., et al. (2013). RP-HPLC fa tongshi ceding Xiasangju keli zhong lvyuansuan, yimidiexiangsuangan, midiexiangsuan he menghuagan [Simultaneous determination of chlorogenic acid，salviaflaside，rosmarinic acid and linarin in Xiasangju Granules by ＲP-HPLC]. *Chinese Traditional Patent Medicine.* 35, 2411-2415.

Liu J. (1995). Pharmacology of oleanolic acid and ursolic acid. *Journal of Ethnopharmacology.* 49, 57-68.

Liu J., Lu Y. F., Wu Q., Xu S. F., Shi F. G., Klaassen C. D. (2019). Oleanolic acid reprograms the liver to protect against hepatotoxicants, but is hepatotoxic at high doses. *Liver International.* 39, 427-439.

Liu W.-g., Su L.-q., Guo X.-l. (2012). Zhongyao Xiasangju fufang tiquwu zhong de huangtong lei chenfen yanjiu [Study on flavonoids in the extract of Chinese Medicine compound Xiasangju]. *International Journal of Traditional Chinese Medicine.* 522-524.

Luyen B. T., Tai B. H., Thao N. P., Lee Y. M., Lee S. H., Jang H. D., et al. (2015). The anti-osteoporosis and antioxidant activities of chemical constituents from Chrysanthemum indicum flowers. *Phytother Res.* 29, 540-8. 10.1002/ptr.5281

Lyu K., Yue W., Ran J., Liu Y., Zhu X. (2021). In vivo therapeutic exploring for Mori folium extract against type 2 diabetes mellitus in rats. *Biosci Rep.* 41. 10.1042/BSR20210977

Ma R., Weng H., Liang J. (2016). Screening of lipase inhibitors in Folium Mori with lipase-linked magnetic microspheres by high-performance liquid chromatography and evaluation in diabetic mice. *J Sep Sci.* 39, 4474-4483. 10.1002/jssc.201600924

Ma W., Wu X.-m., Zhang K.-g. (2011). Xiasangju tiquwu dui ziyouji de qingchu zuoyong [Free radical scavenging effect of Xiasangju extract]. *Chinese Journal of Spectroscopy Laboratory.* 28, 2313-2316.

Ming K., Yuan W., Chen Y., Du H., He M., Hu Y., et al. (2019). PI3KC3-dependent autophagosomes formation pathway is of crucial importance to anti-DHAV activity of Chrysanthemum indicum polysaccharide. *Carbohydr Polym.* 208, 22-31. 10.1016/j.carbpol.2018.12.035

Nakazaki E., Tsolmon S., Han J., Isoda H. (2013). Proteomic study of granulocytic differentiation induced by apigenin 7-glucoside in human promyelocytic leukemia HL-60 cells. *Eur J Nutr.* 52, 25-35. 10.1007/s00394-011-0282-4

Namgung S., Yoon J. J., Yoon C. S., Han B. H., Choi E. S., Oh H., et al. (2017). Prunella vulgaris Attenuates Diabetic Renal Injury by Suppressing Glomerular Fibrosis and Inflammation. *Am J Chin Med.* 45, 475-495. 10.1142/S0192415X1750029X

Nepali S., Cha J. Y., Ki H. H., Lee H. Y., Kim Y. H., Kim D. K., et al. (2018). Chrysanthemum indicum Inhibits Adipogenesis and Activates the AMPK Pathway in High-Fat-Diet-Induced Obese Mice. *Am J Chin Med.* 46, 119-136. 10.1142/S0192415X18500076

Nolkemper S., Reichling J., Stintzing F. C., Carle R., Schnitzler P. (2006). Antiviral effect of aqueous extracts from species of the Lamiaceae family against Herpes simplex virus type 1 and type 2 in vitro. *Planta Med.* 72, 1378-82. 10.1055/s-2006-951719

Ododo M. M., Choudhury M. K., Dekebo A. H. (2016). Structure elucidation of beta-sitosterol with antibacterial activity from the root bark of Malva parviflora. *Springerplus.* 5, 1210. 10.1186/s40064-016-2894-x

Oh C., Price J., Brindley M. A., Widrlechner M. P., Qu L., Mccoy J. A., et al. (2011). Inhibition of HIV-1 infection by aqueous extracts of Prunella vulgaris L. *Virol J.* 8, 188. 10.1186/1743-422X-8-188

Osakabe N., Yasuda A., Natsume M., Sanbongi C., Kato Y., Osawa T., et al. (2002). Rosmarinic acid, a major polyphenolic component of Perilla frutescens, reduces lipopolysaccharide (LPS)-induced liver injury in D-galactosamine (D-GalN)-sensitized mice. *Free Radic Biol Med.* 33, 798-806. 10.1016/s0891-5849(02)00970-x

Park C. M., Song Y. S. (2013). Luteolin and luteolin-7-O-glucoside inhibit lipopolysaccharide-induced inflammatory responses through modulation of NF- kappa B/AP-1/PI3K-Akt signaling cascades in RAW 264.7 cells. *Nutrition Research and Practice.* 7, 423-429.

Park M. Y., Kang D. H. (2021). Antibacterial Activity of Caffeic Acid Combined with UV-A Light against Escherichia coli O157:H7, Salmonella enterica Serovar Typhimurium, and Listeria monocytogenes. *Applied and Environmental Microbiology.* 87.

Park S.-N., Lim Y. K., Cho E., Jo E., Park P.-S., Kook J.-K. (2014). Antimicrobial Activity of Mulberry Leaf against Mutans Streptococci and Periodontopathogens. *International Journal of Oral Biology.* 39, 201-206.

Ponnulakshmi R., Shyamaladevi B., Vijayalakshmi P., Selvaraj J. (2019). In silico and in vivo analysis to identify the antidiabetic activity of beta sitosterol in adipose tissue of high fat diet and sucrose induced type-2 diabetic experimental rats. *Toxicology Mechanisms and Methods.* 29, 276-290.

Psotova J., Kolar M., Sousek J., Svagera Z., Vicar J., Ulrichova J. (2003). Biological activities of Prunella vulgaris extract. *Phytother Res.* 17, 1082-7. 10.1002/ptr.1324

Raafat K., Wurglics M., Schubert-Zsilavecz M. (2016). Prunella vulgaris L. active components and their hypoglycemic and antinociceptive effects in alloxan-induced diabetic mice. *Biomed Pharmacother.* 84, 1008-1018. 10.1016/j.biopha.2016.09.095

Rocha J., Eduardo-Figueira M., Barateiro A., Fernandes A., Brites D., Bronze R., et al. (2015). Anti-inflammatory effect of rosmarinic acid and an extract of Rosmarinus officinalis in rat models of local and systemic inflammation. *Basic Clin Pharmacol Toxicol.* 116, 398-413. 10.1111/bcpt.12335

Ryu S. Y., Oak M. H., Yoon S. K., Cho D. I., Yoo G. S., Kim T. S., et al. (2000). Anti-allergic and anti-inflammatory triterpenes from the herb of Prunella vulgaris. *Planta Medica.* 66, 358-360.

Sheng Y., Zheng S. J., Zhang C. H., Zhao C. H., He X. Y., Xu W. T., et al. (2018). Mulberry leaf tea alleviates diabetic nephropathy by inhibiting PKC signaling and modulating intestinal flora. *Journal of Functional Foods.* 46, 118-127.

Song J., Zhang Z., Hu Y., Li Z., Wan Y., Liu J., et al. (2021a). An aqueous extract of Prunella vulgaris L. inhibits the growth of papillary thyroid carcinoma by inducing autophagy in vivo and in vitro. *Phytother Res.* 10.1002/ptr.7015

Song Y. G., Kang L., Tian S., Cui L. L., Li Y., Bai M., et al. (2021b). Study on the anti-hepatocarcinoma effect and molecular mechanism of Prunella vulgaris total flavonoids. *J Ethnopharmacol.* 273, 113891. 10.1016/j.jep.2021.113891

Song Y. S., Park C. M. (2014). Luteolin and luteolin-7-O-glucoside strengthen antioxidative potential through the modulation of Nrf2/MAPK mediated HO-1 signaling cascade in RAW 264.7 cells. *Food and Chemical Toxicology.* 65, 70-75.

Sun H. X., Qin F., Pan Y. J. (2005). In vitro and in vivo immunosuppressive activity of Spica Prunellae ethanol extract on the immune responses in mice. *J Ethnopharmacol.* 101, 31-6. 10.1016/j.jep.2005.03.023

Sun L. C., Zhang H. B., Gu C. D., Guo S. D., Li G., Lian R., et al. (2018). Protective effect of acacetin on sepsis-induced acute lung injury via its anti-inflammatory and antioxidative activity. *Archives of Pharmacal Research.* 41, 1199-1210.

Sun X., Yamasaki M., Katsube T., Shiwaku K. (2015). Effects of quercetin derivatives from mulberry leaves: Improved gene expression related hepatic lipid and glucose metabolism in short-term high-fat fed mice. *Nutr Res Pract.* 9, 137-43. 10.4162/nrp.2015.9.2.137

Sun Z. L., Zhang X. X., Wu H. H., Wang H. Y., Bian H., Zhu Y. Z., et al. (2020). Antibacterial activity and action mode of chlorogenic acid against Salmonella Enteritidis, a foodborne pathogen in chilled fresh chicken. *World Journal of Microbiology & Biotechnology.* 36.

Tanida I., Shirasago Y., Suzuki R., Abe R., Wakita T., Hanada K., et al. (2015). Inhibitory Effects of Caffeic Acid, a Coffee-Related Organic Acid, on the Propagation of Hepatitis C Virus. *Jpn J Infect Dis.* 68, 268-75. 10.7883/yoken.JJID.2014.309

Thabti I., Albert Q., Philippot S., Dupire F., Westerhuis B., Fontanay S., et al. (2020). Advances on Antiviral Activity of Morus spp. Plant Extracts: Human Coronavirus and Virus-Related Respiratory Tract Infections in the Spotlight. *Molecules.* 25. 10.3390/molecules25081876

Tsuji-Naito K., Saeki H., Hamano M. (2009). Inhibitory effects of Chrysanthemum species extracts on formation of advanced glycation end products. *Food Chemistry.* 116, 854-859.

Utsunomiya H., Ichinose M., Ikeda K., Uozaki M., Morishita J., Kuwahara T., et al. (2014). Inhibition by caffeic acid of the influenza A virus multiplication in vitro. *Int J Mol Med.* 34, 1020-4. 10.3892/ijmm.2014.1859

Vostalova J., Zdarilova A., Svobodova A. (2010). Prunella vulgaris extract and rosmarinic acid prevent UVB-induced DNA damage and oxidative stress in HaCaT keratinocytes. *Arch Dermatol Res.* 302, 171-81. 10.1007/s00403-009-0999-6

Wang C. M., Jhan Y. L., Tsai S. J., Chou C. H. (2016). The Pleiotropic Antibacterial Mechanisms of Ursolic Acid against Methicillin-Resistant Staphylococcus aureus (MRSA). *Molecules.* 21.

Wu H., Gao M., Ha T., Kelley J., Young A., Breuel K. (2012). Prunella vulgaris aqueous extract attenuates IL-1beta-induced apoptosis and NF-kappaB activation in INS-1 cells. *Exp Ther Med.* 3, 919-924. 10.3892/etm.2012.524

Wu J. (2019). Xiasangju youxiao buwei de zongzaogan ceding [Determination of total saponins in the active parts of Xanthium]. *Strait Pharmaceutical Journal.* 31, 95-97.

Wu J., Zhu Y., Li F., Zhang G., Shi J., Ou R., et al. (2016). Spica prunellae and its marker compound rosmarinic acid induced the expression of efflux transporters through activation of Nrf2-mediated signaling pathway in HepG2 cells. *J Ethnopharmacol.* 193, 1-11. 10.1016/j.jep.2016.07.021

Wu W., Liang K. L., Chen B., Su J., Chen S. H., Lyu G. Y. (2017). [Effects of Mori Folium extract on diet-induced obesity mechanism in rats]. *Zhongguo Zhong Yao Za Zhi.* 42, 1757-1761. 10.19540/j.cnki.cjcmm.2017.0069

Xia B.-h., Pi S.-l., Zhou Y.-m., Xie J.-c., Lin L.-m., Li Y.-m. (2016a). HPLC yanjiu Xiasangju keli peiwu chengfen bianhuaguilv [HPLC study on the variation pattern of dosage components of Xiasangju granules]. *Journal of Chinese Medicinal Materials.* 39, 1813-1816.

Xia B.-h., Yan D., Cao Y., Zhou Y.-m., Li Y.-m., Xie J.-c., et al. (2016b). Butong jixing Xiasangju keli HPLC zhiwentupu ji qi moshishibiefenxi [Analysis of different dosage forms of Xiasangju granules on fingerprints and models using high performance liquid chromatography]. *China Journal of Chinese Materia Medica.* 41, 416-420.

Xin Y.-z., Tang W.-z. (2013). Xiasangju fufang zhong duofen lei chenfen de HPLC-MS fenxi [HPLC-MS analysis of polyphenolic components in Xiasangju Compound]. *Shandong Journal of Traditional Chinese Medicine.* 32, 828-830.

Xu Z. F., Sun X. K., Lan Y., Han C., Zhang Y. D., Chen G. (2017). Linarin sensitizes tumor necrosis factor-related apoptosis (TRAIL)-induced ligand-triggered apoptosis in human glioma cells and in xenograft nude mice. *Biomed Pharmacother.* 95, 1607-1618. 10.1016/j.biopha.2017.08.021

Yang M. Y., Wu C. H., Hung T. W., Wang C. J. (2020). Endoplasmic Reticulum Stress-Induced Resistance to Doxorubicin Is Reversed by Mulberry Leaf Polyphenol Extract in Hepatocellular Carcinoma through Inhibition of COX-2. *Antioxidants.* 9.

Yao J.-x., Fang T.-z., Peng T.-h., Ji X.-m., Zhang X.-c., Zhang J.-k., et al. (2017a). Xiasangju zai zhibei fangzhi denggere de yaowu zhong de xinyingyong [New application of Xiasangju in the preparation of drugs against dengue fever]. CHINA Patent CN107397793A. China National Intellectual Property Administration.

Yao J.-x., Fang T.-z., Peng T.-h., Pu Q.-h., Zhang X.-c., Zhang J.-k., et al. (2017b). Xiasangju zai zhibei fangzhi shouzukoubing de yaowu zhong de xinyingyong [New application of Xiasangju in the preparation of drugs against hand, foot and mouth disease]. CHINA Patent CN107412340A. ADMINISTRATION, C. N. I. P.

Youssef F. S., Eid S. Y., Alshammari E., Ashour M. L., Wink M., El-Readi M. Z. (2020). Chrysanthemum indicum and Chrysanthemum morifolium: Chemical Composition of Their Essential Oils and Their Potential Use as Natural Preservatives with Antimicrobial and Antioxidant Activities. *Foods.* 9. 10.3390/foods9101460

Yu F., Zhang L., Ma R., Liu C., Wang Q., Yin D. (2021). The Antitumour Effect of Prunella vulgaris Extract on Thyroid Cancer Cells In Vitro and In Vivo. *Evid Based Complement Alternat Med.* 2021, 8869323. 10.1155/2021/8869323

Yu Q., Li X., Cao X. (2017). Linarin could protect myocardial tissue from the injury of Ischemia-reperfusion through activating Nrf-2. *Biomedicine & Pharmacotherapy.* 90, 1-7.

Yu S.-m., Li Y.-h., Jiang Y., Wang L. (2018). Xiasangju dui jiaxing H1N1 liugan bingdu de zuoyong jiqi jizhi [Effect of XiaSangju on influenza A（H1N1）virus and relevant mechanism]. *Chinese Journal of Biologicals.* 31, 1099-1103.

Zhan H.-q., Dong T.-x. (2009). Yizhong kangliugandu de zhongyao youxiao buwei jiqi zhibei fangfa [An effective part of an anti-influenza virus Chinese medicine and its preparation method]. CHINA Patent CN100493532C.

Zhang L., Yao J.-x., Ji X.-m., Yu T.-t., Pu Q.-h., Tang Ｒ., et al. (2019a). Xiasangju keli tiwai kang Ⅰxing denggere bingdu de zuoyong Inhibitory effects of Xiasangju granules on dengue virus type Ⅰ in vitro]. *Guangdong Medical Journal* 40, 1250-1254.

Zhang X., Ao Z., Bello A., Ran X., Liu S., Wigle J., et al. (2016). Characterization of the inhibitory effect of an extract of Prunella vulgaris on Ebola virus glycoprotein (GP)-mediated virus entry and infection. *Antiviral Res.* 127, 20-31. 10.1016/j.antiviral.2016.01.001

Zhang X., Wu J. Z., Lin Z. X., Yuan Q. J., Li Y. C., Liang J. L., et al. (2019b). Ameliorative effect of supercritical fluid extract of Chrysanthemum indicum Linnen against D-galactose induced brain and liver injury in senescent mice via suppression of oxidative stress, inflammation and apoptosis. *J Ethnopharmacol.* 234, 44-56. 10.1016/j.jep.2018.12.050

Zhen Z. G., Ren S. H., Ji H. M., Ma J. H., Ding X. M., Feng F. Q., et al. (2017). Linarin suppresses glioma through inhibition of NF-kappa B/p65 and up-regulating p53 expression in vitro and in vivo. *Biomedicine & Pharmacotherapy.* 95, 363-374.

Zheng Q. S., Sun X. L., Xu B., Li G., Song M. (2005). Mechanisms of apigenin-7-glucoside as a hepatoprotective agent. *Biomedical and Environmental Sciences.* 18, 65-70.

Zheng X. Q., Song L. X., Han Z. Z., Yang Y. B., Zhang Y., Gu L. H., et al. (2022). Pentacyclic triterpenoids from spikes of Prunella vulgaris L. with thyroid tumour cell cytostatic bioactivities. *Nat Prod Res.* 1-9. 10.1080/14786419.2021.2024532

Zhou F.-f. (2012). Xiasangju fufang huaxue chengfen de yanjiu [Study on Chemical Constituents of Xiasangju Formula]. Master Master, Jinan: University of Jinan.

Zhou F.-f., Tang W.-z., Wang X.-j., Jia X.-h. (2012). Xiasangju fufang huaxue chengfen de yanjiu [Study on Chemical Constituents of Xiasangju Formula]. *Food and Drug.* 14, 107-109.

Zhou Q. X., Liu F., Zhang J. S., Lu J. G., Gu Z. L., Gu G. X. (2013). Effects of triterpenic acid from Prunella vulgaris L. on glycemia and pancreas in rat model of streptozotozin diabetes. *Chin Med J (Engl).* 126, 1647-53.

Zhu J., Zhang W., Zhang Y., Wang Y., Liu M., Liu Y. (2018). Effects of Spica prunellae on caspase-3-associated proliferation and apoptosis in human lung cancer cells in vitro. *J Cancer Res Ther.* 14, 760-763. 10.4103/jcrt.JCRT_1289_16

Zhu Q., Muyayalo K. P., Xu Q. H., Wang J., Wang H., Liao A. H. (2022). Prunella vulgaris can improve the pregnancy outcomes of experimental autoimmune thyroiditis rats by inhibiting Th1/Th17 immune responses. *J Reprod Immunol.* 149, 103469. 10.1016/j.jri.2021.103469
